# An Insight into Ionic Conductivity of Polyaniline Thin Films

**DOI:** 10.3390/ma13122877

**Published:** 2020-06-26

**Authors:** Pavel Chulkin, Mieczysław Łapkowski

**Affiliations:** 1 Department of Physical Chemistry and Technology of Polymers, Faculty of Chemistry, Silesian University of Technology, Strzody 9,44-100 Gliwice, Poland; pavel.chulkin@polsl.pl; 2 Centre of Polymer and Carbon Materials of the Polish Academy of Sciences, MariiSkłodowskiej-Curie 34, 41-819 Zabrze, Poland

**Keywords:** EIS, EQCM, conductive polymer, polyaniline, ionic conductivity

## Abstract

The work addresses an issue of the conductivity phenomenon in conductive polymer thin films. Polyaniline was chosen as a broadly used and thoroughly investigated conductive polymer in order to test and show capabilities of the developed original approach based on impedance spectra analysis. A number of films of different thickness were deposited onto a Pt electrode surface and consequently investigated in aqueous solution containing perchloric acid as an electrolyte. The processes that occur in polyaniline film were studied by cyclic voltammetry, electrochemical quartz crystal microgravimetry (EQCM) and electrochemical impedance spectroscopy (EIS). The role of incorporated ions as charge carriers was investigated with respect to the control of the conductivity properties of the film. Along with detailed polyaniline behavior study, the work makes up a fundamental scientific impact on theoretical electrochemistry and electroanalytical techniques.

## 1. Introduction

The electrochemistry of conductive polymers has remained an object of interest for the last several decades for several reasons including the unique properties of this type of material, the diversity of its application, and the inexhaustible possibility of its structure modification. The currently developing organic electronics area raised conductive polymers to the top perspective on materials covering a wide range of applications [1,2]. Although the target application of a polymer is concerned with solid state, prior electrochemical and spectroelectrochemical characterization remain helpful for predicting material performance [3]. Mechanisms of charge transport in conductive polymers are known generally, however there is still neither conventional quantitative data describing these aspects, nor a universal strategy for charge transfer parameters estimation.

Conductivity is not a constant characteristic of the material. It is a complicated function of basic charge transport parameters: charge density and mobility. Real systems include more than one type of mobile charge carrier and their density and mobility are functions of the applied electric field. Therefore, in the case when a semiconducting system is far from ideal (i.e., isotropic and trap-free), the creation of the theoretical model is an incredibly complicated challenge.

In order to understand the conductivity performance of a material, one has to clearly distinguish between direct current (DC) and alternating current (AC) conductivity phenomena. The alternative current requires mobile charged species which are able to move providing oscillation of space charge. However, if a constant voltage is applied, the space charge at the contact immediately becomes saturated and no charge flow occurs. For instance, a water solution containing sodium chloride is a typical ionic conductor. Only if the voltage is increased significantly, does the electrolysis start and the constant DC current is maintained. The ionic conductor needs electroactive species that would be able to accept or give charge through the interface, so that constant direct current is able to pass. Thus, all DC-conductive materials are also AC-conductive, but AC conductivity is not always evidence of DC conductivity. In other words, apparent high conductivity measured by AC signal-based technique gives no proof about high DC conductivity and its type (ionic or electronic). The objective of the current article is to take up questions related to nature of charge transport in conductive polymers and the ways of monitoring it.

Polyaniline, being one of the first discovered [4] and most popular representatives of conductive polymers [5], remains a convenient object for testing new techniques and investigation of effects concerning conductive polymers. Polyaniline’s electrochemical [6,7,8] and spectroelectrochemical [9,10] investigation along with other aspects including intermediates formed during electropolymerization [11], synthesis conditions influence [12], effect of doping ions [8,13,14,15,16], polaron–bipolaron states [17,18], isotope effects [19], memory effect [20] and advanced applications [21,22] were an object of research carried out in research groups of Genies and Lapkowski in the last two decades of the 20th century.

Electrochemical impedance spectroscopy (EIS) [23] as an advanced electrochemical method is useful for in depth study of charge transport, as it allows for separation of AC current signal into signals of single stages of charge transport or system structural elements due to their different frequency response. In previous years, the researchers undertook many attempts to fit the experimental impedance spectrum by different equivalent electrical circuits consisting of several physical elements [24,25,26,27]. Authors of the works [28,29,30,31] were employing a modified diffusion impedance (Warburg) element to equivalent electrical circuit, taking into account a hindered diffusion of charge carriers and considering polymer film as a porous conductive medium. The most fundamental theoretical works were performed by Vorotyntsev [32,33], Láng and Inzelt [33,34,35] and Bisquert [36,37]. The authors considered all the factors including potential distribution and electro active species diffusion and migration and carried out simulation of impedance spectra. However, even the most sophisticated theoretical approach cannot take into consideration all the defects and other non-ideality factors that indispensably take place in a polymer film. All the simulation techniques are based on equations that were derived for ideal case. Sometimes empirical coefficients are added in order to approximate theory to experiment, although such coefficients have no physical sense and cannot be used for estimation of system physical parameters. Until now, there has been no generally recognized, universal methodology for impedance characterization of conductive polymers.

In this work we aime dour efforts at the maximization of direct interpretation of experimentally obtained data, trying to minimize expectations and predictions based on idealized simulation models. The unavoidable approximations are mentioned in the text. The target parameters for estimation were concentration of charged species and their diffusion coefficients.

## 2. Materials and Methods

Polyaniline film was deposited onto an electrode surface from aqueous solution containing 0.2 M aniline (purchased from Sigma Aldrich, Merck group, St. Louis, MO, USA) and 1.0 M HClO_4_ (purchased from POCH S. A., Avantor Performance Materials Poland, Gliwice, Poland) by cycling electrode potential within the range from −0.2 V to +1.1 V vs. AgCl/Ag covering oxidation and reduction peaks. Electrochemical characterization of the obtained polymer film was carried out in the narrower potential range from 0.1 V to 0.8 V. This range was chosen in order avoid destruction of the film by oxidation or reduction. Within this range, the film was found to be stable for a long exposure time required for impedance spectrum registration. Investigation of the deposited polymer film was carried out in aqueous solution containing 0.1 M HClO_4_. The solution was deaerated for 20 min before measurement and during the experiment by bubblin gargon inside the cell above the solution.

The electrochemical characterization was accomplished using a BioLogic SP-150 (BioLogic Science Instruments, Seyssinet-Pariset, France) potentiostat with a built-in frequency response analyzer. Pt working electrode ET075 from eDAQ (eDAQ Pty Ltd, Denistone East, Australia), and a Pt counter electrode (wire) and Ag/AgCl reference electrode ET069 from eDAQ were employed in a three-electrode 3 mL eDAQ electrochemical cell. The counter electrode was flame annealed and the working electrode was polished before each experiment. Electrochemical quartz crystal micro gravimetry (EQCM) was carried out using a CHi 400C potentiostat (CH Instruments, Inc., Austin, TX, USA) with time-resolved electrochemical quartz crystal microbalance (EQCM) module. Potentiodynamic electrochemical impedance spectra (PDEIS) were obtained using a home-made system [38] including ПИ-50 potentiostat (PI-50, Factory of measuring instruments and computer engineering, Orenburg, Russia), computer synchronized via analog-to-digital transformer and special software.

The potential scan rate used in cyclic voltammetry was 100 mV/s. Stationary impedance spectra were obtained in the1 Hz–100 kHz frequency range with 20 points per decade in a logarithmic scale (total number of frequencies in one spectrum was 101). In order to achieve stationary conditions, the potential was maintained for 1 min after the potential switch, before each spectrum registration. PDEIS frequency range was from 14 Hz to 439 Hz.

Analysis of electrochemical impedance spectra, determination of equivalent circuit parameters and other calculation procedures were realized using Microsoft Excel 2013 software (Microsoft, Redmond, WA, USA). Complicated mathematical derivation procedures in analytic form were accomplished in the Wolfram Mathematica 11 program.

## 3. Results

### 3.1. Cyclic Voltammetry of the Polyaniline Film

**Figure 1:**
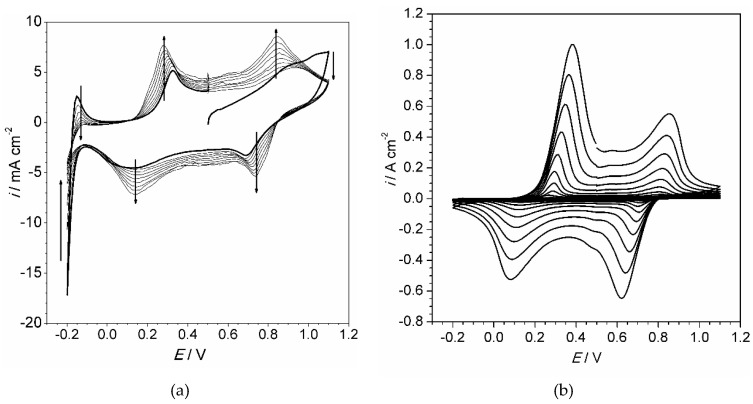

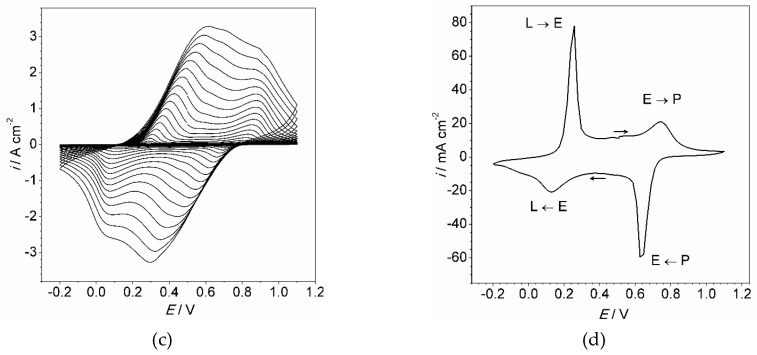
Cyclic voltammograms of aniline electropolymerization (**a**–**c**): 10 cycles (**a**), 100 cycles (**b**) and 200 cycles (**c**) (each tenth cycle is shown); cyclic voltammogram of polyaniline film (obtained by 50 cycles) in acidic aqueous solution (**d**). Aniline concentration 0.2 M (**a**–**c**), HClO_4_ concentration 1 M, scan rate 0.1 V·s^−1^, dichloromethane solution. All potentials vs. AgCl/Ag reference electrode. Vertical arrows (**a**) indicate peak evolution during scanning. Labels (**d**) attribute peak to the corresponding transformation between polyaniline forms.

The cyclic voltammograms characterizing the polyaniline electrodeposition procedure are shown in Figure 1.

Regarding the oxidation state, polyaniline exists in three forms: the neutral form leucoemeraldine (L), the partially oxidized form emeraldine (E) which is conductive, and the utmost oxidized form pernigraniline (P) [1]. Although further oxidation is possible, this causes destruction of a conjugated chain and loss of conductivity, therefore high positive potential range is not considered. The mechanism of polyaniline oxidation and growth of polyaniline film is presented in Appendix A.

### 3.2. Study of the Aniline Electrochemical Polymarization by Electrochemical Quartz Crystal Microgravimetry(EQCM)

**Figure 2:**
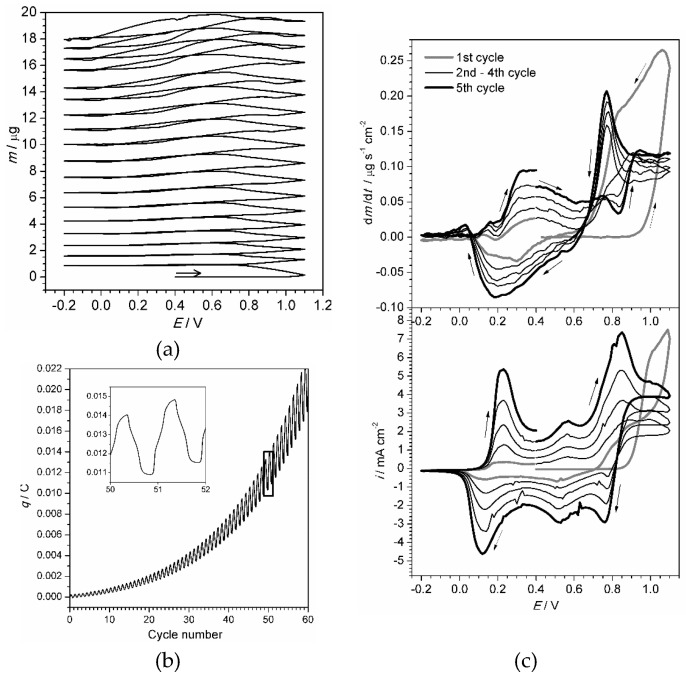
(**a**): Voltmassogram of aniline electropolymerization during first 15 cycles. (**b**) Dependence of consumed charge on time during cyclic electropolymerization. (**c**) Comparison of differential voltmassogram and voltammogram for first five cycles of electropolymerization. Aniline concentration 0.2 M, HClO_4_ concentration 1 M, scan rate 0.1 V·s^−1^, aqueous solution. All potentials vs. AgCl/Ag reference electrode. Electrode surface area 0.205 cm^2^.

The electrochemical quartz crystal microgravimetry (EQCM) method has been applied to obtain direct evidence of the growth of polymer film and to relate the film mass to the consumed electrical charge. Figure 2 shows a voltmassogram registered for aniline electropolymerization.

The term “voltmassogram” is not common in literature, due to low dissemination of the EQCM technique. However, since there is no other equivalent denomination, we regard the term “voltmassogram” as a convenient and concise description for the EQCM results.

### 3.3. Characterization of the Polyaniline Film by Impedance Spectroscopy

**Figure 3:**
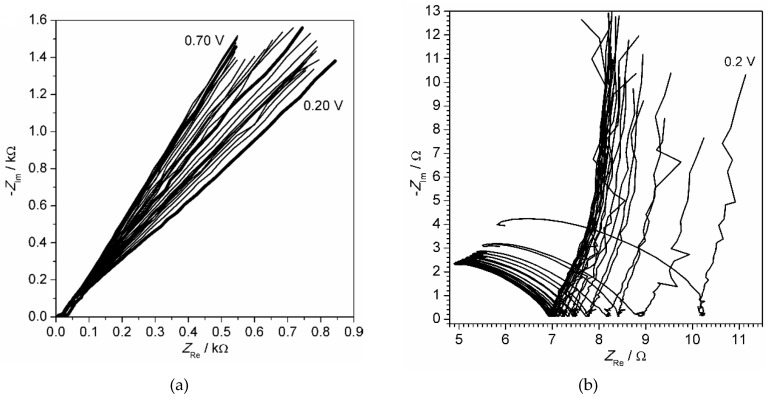
Impedance spectra of polyaniline film obtained by 10 cycles (**a**) and 100 cycles (**b**) of electropolymerization. The spectra registered within the potential range from 0.2 V to 0.7 V with 0.02 V increment.

**Figure A1:**
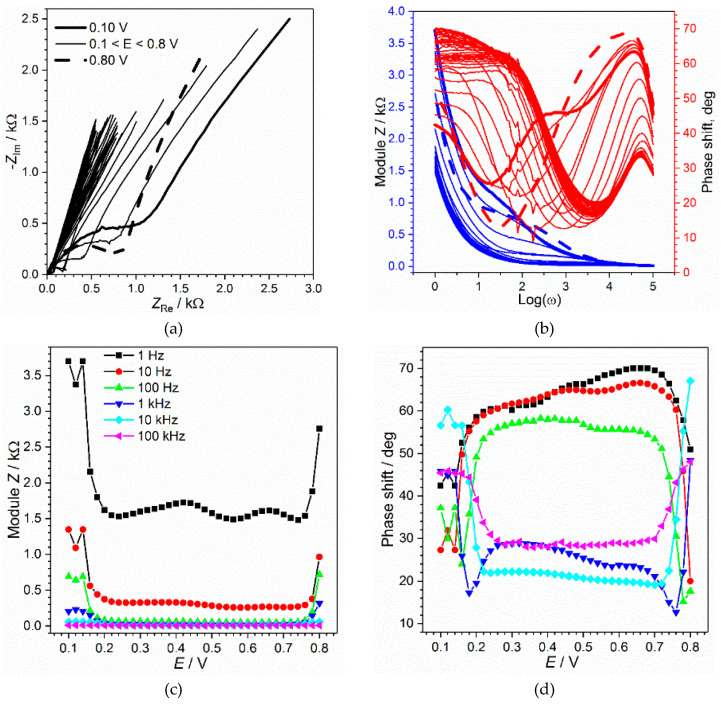
Set electrochemical impedance spectra of polyaniline film obtained by 10 cycles of electrodeposition within the potential range 0.1 V–0.8 V in 0.1 M HClO_4_ aqueous solution: complex plane (Nyquist) plot (**a**) and Bode plot (**b**). Dependence of impedance module (**c**) and phase shift (**d**) on potential for chosen AC frequencies.

**Figure A2:**
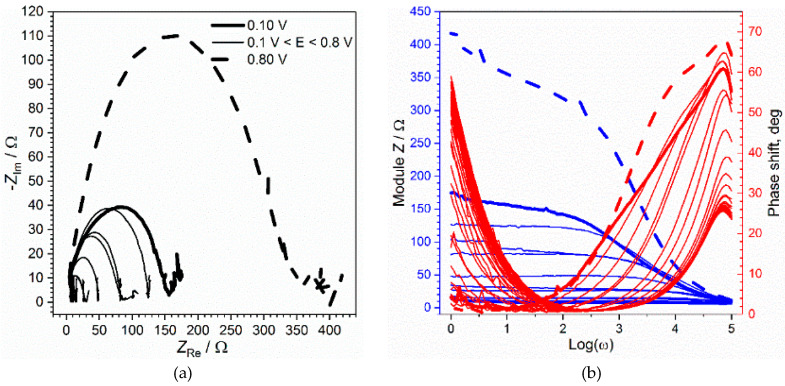

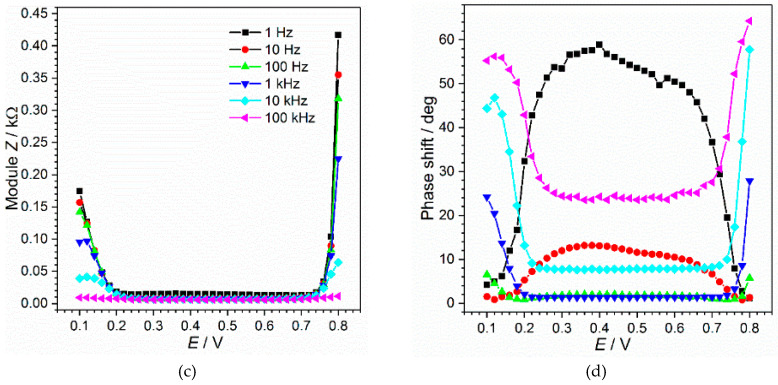
Set electrochemical impedance spectra of polyaniline film obtained by 100 cycles of electrodeposition within the potential range 0.1 V–0.8 V in 0.1 M HClO_4_ aqueous solution: complex plane (Nyquist) plot (**a**) and Bode plot (**b**). Spectra corresponding to left potential scan interval border (0.1 V) and right border (0.8 V) are highlighted by thick and thick dashed line, respectively. Dependence of impedance module (**c**) and phase shift (**d**) on potential for chosen AC frequencies.

The output results obtained by the electrochemical impedance spectroscopy, the most complicated and informative method employed in the current work, are shown in Figure 3, Figure A1 and Figure A2 (Appendix B). They illustrate a raw data obtained for the thinnest (10 electropolymerization cycles, Figure 3a and Figure A1) and thickest (100 electropolymerization cycles, Figure 3b and Figure A2) polyaniline film. Each complex set of results included 70 impedance spectra, each of them consisting of 101 points. Each resulting point is characterized by three parameters: AC frequency (ω), real impedance (*Z*_Re_) and imaginary impedance (*Z*_Im_). The last two values are shown in complex plane plot, also called the Nyquist plot (Figure 3a,b). Additional information can be acquired from impedance module (│*Z*│) (Figure A1b,c and Figure A2b,c) and phase shift (ϕ) (Figure A1b,d and Figure A2b,d). Although the latter values can be derived from the aforementioned *Z*_Re_ and *Z*_Im_, they allow us to observe tendencies along the frequency scale and to compare wide range data on a single plot.

**Figure 4:**
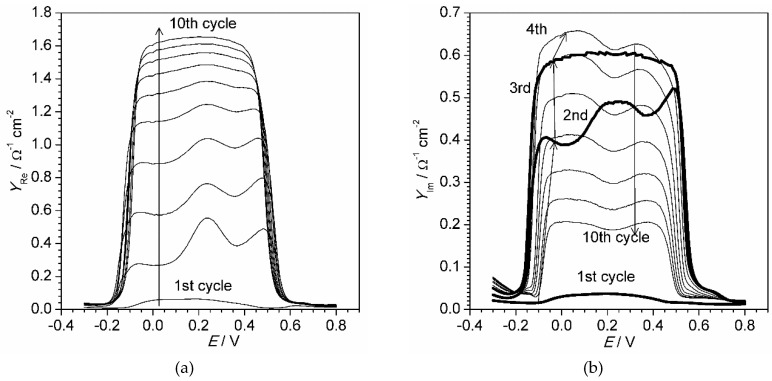
Real (**a**) and imaginary (**b**) admittance of aniline polymerization registered in the dynamic mode at 10 mV·s^−1^ at frequency 60 Hz.

The classical potentiostatic impedance technique is eligible for monitoring stationary processes and objects. In order to observe conductivity change during electropolymerization a potentiodynamic electrochemical impedance spectroscopy was used. It allows fast registration of impedance spectra, yet the frequency range is limited. Figure 4 demonstrates evolution of polyaniline film complex admittance (inverse of impedance) components during the first 10 scans of cyclic electropolymerization.

## 4. Theoretical Part

Analysis of impedance spectra is traditionally accomplished by a search for equivalent electric circuit. The most profound solution includes not only finding the circuit but also its theoretical justification that allows attributing a physical sense of each elementary parameter. Numerous attempts to find an adequate equivalent electrical circuit that could fit experimental data in the whole frequency range were not successful. Rather simple circuits, including those claimed before [24,25,26], could be used in narrow frequency ranges. The repetitive failure encouraged us to advance another approach based on theoretical knowledge about charge transport in semiconductors and solutions. The objective was to develop a technique capable of estimating real physicochemical properties of the polymer, such as the concentration and mobility of charged species. One of the most important factors to be taken into account was electric field distribution and oscillation. The electric field gradient along with its oscillation under AC conditions is usually neglected when the electrode–solution interface is considered. The assumption is reasonable, provided that electrical double layer is very thin and does not affect Faradaic process and migration of electroactive species. However, in the case of polymer film, consideration of electric field oscillation appeared to be essential to obtain a suitable theoretical model.

To derive the final formula one has to (i) consider oscillating parameters affecting current, (ii) represent current oscillation as a function of them, (iii) express all the parameters in terms of oscillating current and voltage and (iv) rearrange the final formula to eliminate the relation of oscillating voltage and current, i.e., impedance [39].

The description of conductive polymer film required consideration of the presence of the potential gradient along the film. For many electrochemical systems discussed in the literature the potential gradient has been taken into account only in the double electric layer region. Its effect was restricted to the region between the electrode surface and outer Helmholtz plane, which comprises the ions that are closest to the electrode surface, but are not specifically adsorbed. Disregarding potential gradient is not reasonable in the case of polymer film. This factor was taken into account in last works on conductive polymer impedance study by Láng and coll [34,35].

**Figure 5:**
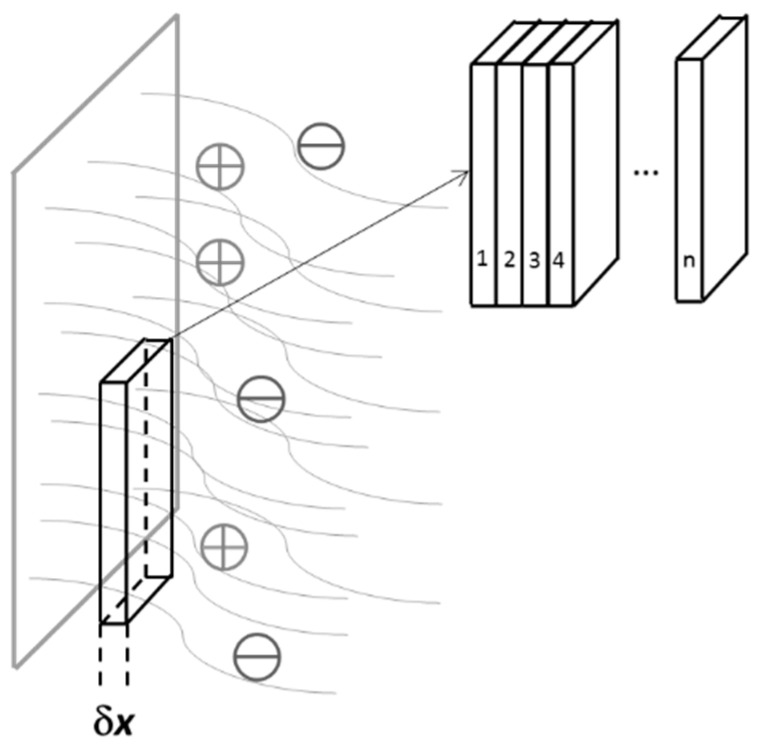
Illustration of mathematical approach that involves analysis of infinitesimal layer and consequent summation (integration) along the whole film thickness.

**Figure 6:**
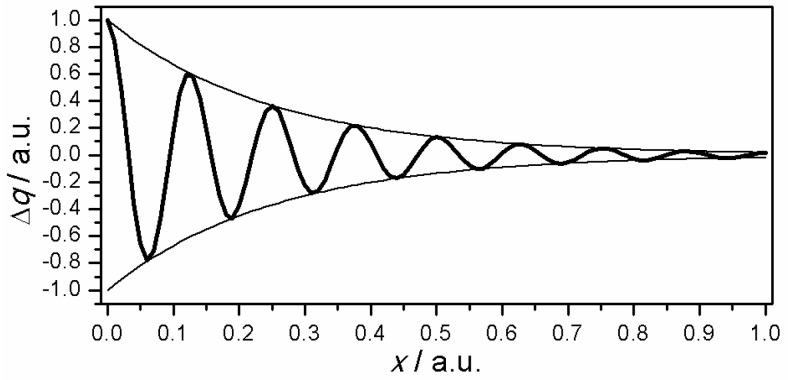
Attenuation of the signal amplitude illustrated as charge oscillation along the distance from the electrode. The schematic illustration is based on the theoretical approach employed.

The complete mathematical processing is explained in Supplementary Materials (Figures S1–S7, Equations (S1–S60)) in details. The polymer film was regarded as a set of discrete ultrathin layers (Figure 5). The AC signal damping has to be taken into account in order to explain observed distortion of the impedance spectrum with changing frequency. According to developed theory the penetration ability of a signal depends on its frequency (Figure 6). The low frequency signal probes thicker volume of the film than the high-frequency signal. Therefore, the remote layers have a larger impact in the total impedance measured at lower AC frequency than in the impedance measured at higher frequency. The discussed phenomena are explicitly described by mathematical formulas below.

The resulting formula for impedance of polymer layer with one type of charge carriers is given below:

(1) $$Z\left(\omega \right)=\underset{k}{\sum }\left(\frac{1}{z^{3}f^{2}FDE\cdot c\left(x\right)}+\frac{1}{j\omega z^{2}fFd\cdot c\left(x\right)}\right)\gamma \left(\omega ,x\right)$$

where *n* is the number of discrete layers taken into consideration, *z* is an elementary charge of the charge carrier (typically +1 or −1), *f* is a parameter substituting expression *F*/*RT* for simplification (V^−1^), *F*—Faraday constant (96,485 C·mol^−1^), *D*—diffusion coefficient (m^2^·s^−1^), *E*—electric field intensity (potential gradient, V·m^−1^), *c*(*x*)—concentration of charged species (mol·m^−3^), ω—AC signal angular frequency (Hz), *d*—thickness of a discrete layer, γ(ω,*x*)—a damping coefficient describing impact of layer located at distance *x* in the impedance measured at frequency ω in Equation (2).

(2) $$\gamma \left(\omega ,x\right)=e^{-\sqrt{\frac{\omega }{2D}}x}$$

If one consolidates all time-independent terms in constants the Equation (1) appears as:

(3) $$Z\left(\omega \right)=\underset{n}{\sum }R+\frac{1}{j\omega C}$$

In the case of the presence of two or more types of charged species, the impedances ensured by them are summarized electrical elements connected in parallel. Thus:

(4) $$Z_{tot.}^{-1}=\overset{k}{\underset{i=1}{\sum }}Z_{i}^{-1}$$

where *k* is number of mobile charged species (charge carriers).

**Figure 7:**
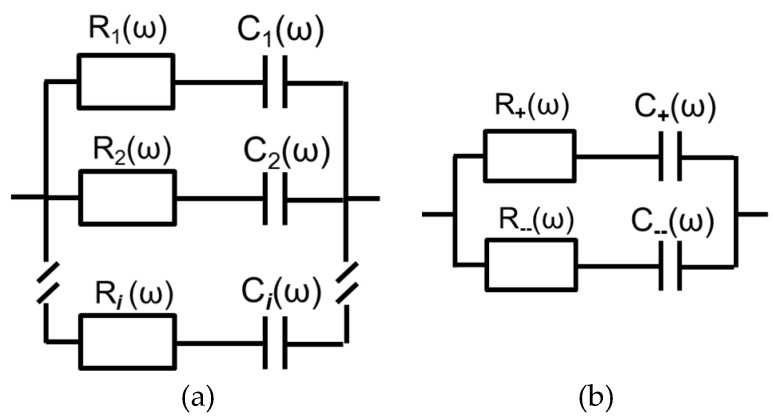
Equivalent circuits corresponding to the worked theoretical model: in general case (**a**) and in the considered work (**b**).

Equation (3) describes an equivalent electric circuit which comprises a resistor and a capacitor connected in series. In case of the presence of several charge carriers, their effect on current would be summarized. That means that the model described by Equation (4) would contain several parallel branches, each corresponding to certain type of ion (Figure 7a). During analysis of experimental data it was found that two branches (Figure 7b) are enough to fit the spectrum. Of course, the presence of additional branches would only improve the fitting, but there is no evidence of necessity of those additional parameters.

The fact that the parameter values are dependent on frequency may be confusing and non-consistent with the conventional concept of equivalent circuits. A similar technique for fragmentary analysis has been already proposed by Vladikova et al. [40,41,42] in respect of the non-uniform research objects. In defense of our idea we recall that the conventional approach of fitting equivalent circuit in the whole range of frequencies could not be applied to the conductive polymer film, and so this was the reason to develop the advanced technique.

According to our hypothesis, one branch of the equivalent circuit (Figure 7b) is attributed to positively charged ions (cations) and the other to negatively charged ions (anions). Afterwards, the attributions will be confirmed by attentive analysis of the results. In the first surprise, for each spectrum one of the resistances appeared to be positive and the other to be negative, while both capacitances remained positive. At the early stages of the work, such results were regarded as erroneous, but their reproducibility in all the samples and under all conditions was convincing enough to accept them and develop an appropriate explanation. The answer to this paradox is hidden in Equation (1), The only term that could be either positive or negative is *z* value in an odd degree. The sign of *z* value defines the sign of *R*, whereas according to Equation (1), *C* is always positive, regardless of *z* due to the fact that *z*^2^ > 0.

A close look at the formulas would perceive that two equations contain four unknown variables (*c*, *D*, *E* and *d*), which makes the problem unsolvable. However, the experimental data for the research object contain manifold points.

The proposed strategy will be aimed at the estimation of resistances and capacitances of each of the infinitesimal layer (Figure 7). The number of the regarded semi-discrete layers may be less than or equal to the number of frequency points, but not exceed that number. Taking into account that precision of the results requires minimization of the thickness of the small layer, we use the maximal possible number, i.e., the number which is equal to the number of frequency points. The procedure includes the following steps.

1. The initial approximate values of diffusion coefficients for cations (*D*_+_) and anions (*D*_−_) are proposed, e.g., 1·× 10^−10^ m^2^·s^−1^. The values will be further adjusted during iteration steps.
2. The thickness of the polymer film is divided by *n* equal fragments (*d* = *l*/*n*), *n* equals number of frequency.
3. The matrix of γ-coefficients Γ*_nn_* is created. Each element is calculated by the Equation (5), which is formed from Equation (2) to estimate the average value of γ in the considered distance interval [*x*_1_; *x*_2_] (see Equation S56 for details).
  (5) $$\gamma \left(\omega ,x_{1},x_{2}\right)=\sqrt{\frac{2D}{\omega }}\frac{1}{x_{2}-x_{1}}\left(e^{-\sqrt{\frac{\omega }{2D}}x_{1}}-e^{-\sqrt{\frac{\omega }{2D}}x_{2}}\right)$$
4. Two systems of equations have to be solved to estimate values of resistances and capacitances of each elementary layer (*R*(*x_i_*) and *C*(*x_i_*)). The system of equation concerned with resistances is shown below in a matrix form.
  The resistance estimated at a given frequency *ω_i_* comprises resistances of *k* film fragments. Due to different γ-coefficients impact of the close fragments to the total resistance is larger than that of the remote fragments:
  (6) $$R\left(\omega_{j}\right)=\overset{k}{\underset{i=1}{\sum }}\left(\gamma \left(\omega ,x\right)R\left(x_{i}\right)\right)$$
  or written in a full matrix form in Equation (7)
  (7) $$\left(\begin{array}{c} R\left(\omega_{1}\right)\\ \ldots \\ R\left(\omega_{j}\right) \end{array}\right)=\left(\begin{array}{ccc} \gamma \left(\omega_{1},x_{1}\right) & \ldots & \gamma \left(\omega_{1},x_{i}\right)\\ \ldots & \ldots & \ldots \\ \gamma \left(\omega_{j},x_{1}\right) & \ldots & \gamma \left(\omega_{j},x_{i}\right) \end{array}\right)\times \left(\begin{array}{c} R\left(x_{1}\right)\\ \ldots \\ R\left(x_{j}\right) \end{array}\right)$$
  The *R*(*x_j_*)—matrix sought could be calculated using inverse matrix approach (in case of equality of number of frequency points and fragments) or by linear function analysis.
  The same approach is applied to estimate values of capacitances.
5. Having the values of capacitances, resistances and diffusion coefficients at one’s disposal means one can estimate the values of concentration in all the considered fragments.
  (8) $$c\left(x_{i}\right)=\frac{C\left(x_{i}\right)}{fFd}$$
  (9) $$E=\frac{1}{zf^{2}FDc\left(x_{i}\right)R\left(x_{i}\right)}$$

The values of electric field obtained for both types of ions have to be the same. That equality makes a criterion for adjusting the diffusion coefficients. The steps 1–5 are repeated until consistency is achieved.

## 5. Discussion

### 5.1. Cyclic Voltammetry

The polymer film occupies the electrode surface after the first cycle of electropolymerization, so all following cycles resemble each other, except the first one (Figure 1a). The high anodic current at potential higher than 1.0 V is observed only at the first scan and is attributed to the oxidation of the monomer molecules on the bare electrode surface. Cathodic and anodic peaks that appear in a potential range at c.a. −0.15 Vare related to hydrogen evolution and oxidation on a platinum electrode. As the polymer film covers electrode surface, hydrogen evolution is suppressed. Two anodic and two cathodic peaks within the range 0.1–0.9 V must be attributed to the growing polymer film. They steadily grow during whole electropolymerization period (Figure 1a–c). Those peaks are clearly observed when polymer film is tested in monomer-free solution (Figure 1d). The voltammogram represents two couples of peaks, each couple corresponding to transformation between polyaniline forms (Appendix A).

### 5.2. EQCM Study

The voltmassogram depicts an expected tendency of successive mass growth caused by bonding of new monomer units to the polymer. The first 15 cycles demonstrate a stable growth with a constant increment which estimated value was equal to 5.3 μg·cm^−2^·cycle^−1^. However, the mass value could not be used for precise estimation of thickness and mass of the polymer film due to eventual absorption of ions and solvent molecules.

We admit that the concept of thickness is not appropriate for polymer film immersed in solution. Nevertheless, a geometrical parameter was necessary to normalize the values, obtained by processing of the experimental data, required for proceeding to physical parameters of charge transport. For that purpose, the layer thickness was approximately evaluated based on the oxidation charge required for electropolymerization. The latter value, if not returned directly by potentiostat, can be calculated by integration of the current by time. Figure 4b shows the plot of charge vs. number of electropolymerization cycles. Table 1 gives the values of charge and estimated thickness for the samples that were repeatedly investigated within the scope of the work.

The values of thickness were calculated based on the Equation (10) between charge and mass of the deposited compound (the Faraday law). The density, to a certain degree of likelihood, was accepted to be 1g·cm^−3^, number of electrons per one monomer unit (*z*) equals two.

*d* = *M*·*q*/(*z*·*F*·*S*·ρ) (10)

Concerning Figure 2b, the positive growth of charge with time confirmed that the polymerization occurs by oxidation, i.e., the total amount of electrons coming from the solution to the electrode (anodic current) is greater than the amount of electrons flowing in opposite direction (cathodic current). The current is not related only to the oxidation of new adding monomer units, but also reflects repetitive oxidation/reduction of the polymer film and absorption/desorption of ions from the solution. The differential voltmassogram is shown in Figure 2c. It illustrates the rate of mass change with comparison to the registered current. During the anodic scan an intensive mass increase starts after the second anodic peak is reached (c.a. +0.85 V) and continues on a backward scan till 0.65 V. The maximum mass increase rate becomes apparent as a sharp maximum at 0.8 V, correlating with first backward scan cathodic current peak. The cathodic scan within the range from 0.6 V through 0.1 V is accompanied by decrease of mass. However, an almost equal amount of mass is returned within the same potential range during the following anodic scan. The observed mass changes are related to electroabsorbtion of ions from solution facilitated by higher conductivity of polyaniline film (emeraldine form). During the anodic scan, the film is positively charged, so that heavy perchlorate anions intercalate into the film. The subsequent reduction of the film (cathodic scan) eliminates its positive charge, and makes the absorbed anions leave the film.

### 5.3. Electrochemical Impedance Spectroscopy (EIS)Study

One of the challenges issued by conductive polymer film is a noticeable change of spectrum with film thickness: the almost straight line (Figure 3a) is transformed to a looped semicircle with a low-frequency tail (Figure 3b). The typical impedance spectrum elements, such as straight line related with a diffusive regime or semicircle related to a Faradaic process do not appear. One may mistakenly recognize simple basic elements, if the scale is ignored. Another problem is extremely high complexity of the spectra caused by non-uniformity and high porosity of the randomly oriented bundle of macromolecules attached to the electrode surface.

The potential dependence plots indicate a decrease of impedance module within the middle scanning potential range from 0.1 to 0.7 V (Figure A1c and Figure A2c) reaching its minimum at 0.42 V. This range coincides with the valley interval between cyclic voltammogram peaks (Figure 1d) and is attributed to an emeraldine form known for its conductivity. The potential dependence of phase shift (Figure A1d and Figure A2d) for thin and thick polymer film is interesting. Very low values of phase shift (about 0°) indicate major resistive behavior of polymer film, whereas high values (about 90°) suggest that capacitive properties prevail. Figure A1d shows that transition to capacitive from resistive behavior occurs when AC signal frequency exceeds 1 kHz. When the film is thicker (Figure A2d), the analogical transition takes place in the lower frequency range between 10 and 100 Hz. The observed effect drew us to the hypothesis that the geometrical size of the probed range may be a function of modulating frequency. The hypothesis has been further developed and confirmed by other facts that arose from analysis of the results.

In Figure 4 we showed the admittance instead of impedance in order to reveal clearly the most important changes in the film complex conductivity. Both real and imaginary admittance demonstrate a high plateau within the potential range between 0.2 and 0.6 V. The shown graph confirms high conductivity properties of the polyaniline film within the defined potential range. The real admittance component increases with growth of the polymer film. The effect is probably related to the increase of the mobile charge carriers and amount of redox compound (conductive polymer) on the electrode surface. The imaginary admittance component increases during the first four cycles of polyaniline deposition, but starts dropping during further growth of the film. The imaginary component value is related to the capacitive properties of the film. Therefore, the following rough explanation of the effect could be given. As soon as polyaniline film appears on the electrode surface, it facilitates absorption of ions and therefore acts as an accumulator of absorbed ions, increasing electrical capacitance of the near-electrode layer. When the film becomes considerably thicker, the electrode potential oscillation has no more effect on ions located near the film-solution border, thus an electrical capacitance becomes smaller like the capacitance of a thick double electric layer.

### 5.4. Analysis of Impedance Spectra

**Figure 8:**
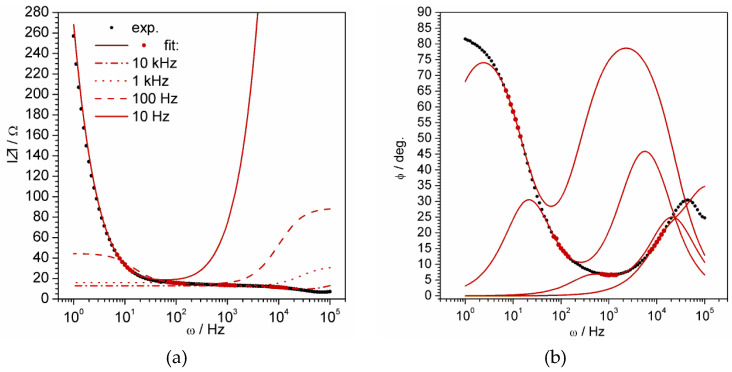
Fitting of the experimental spectrum fragments by equivalent electric circuit shown in Figure 9b: impedance module (**a**) and phase shift (**b**). The demonstrated fitted fragments (red points) included seven points within proximity to 10 Hz, 100 Hz, 1 kHz, 10 kHz. The spectrum was registered for 10 cycle-deposited polyaniline film at 0.42 V.

Multiple attempts to find the best solution revealed a very interesting phenomenon that appeared to be a unique and excellent solution of the problem. It was observed that equivalent circuit derived for a model thin layer (Figure 7b) can ideally fit experimental data in a narrow frequency range (width of about one order of frequencies). The only problem was non-equality of parameters of resistance and capacitance in different frequency ranges. The reasonability of fragmental analysis of impedance spectra has been precisely regarded by Vladikova and co-workers [40,41,42] with particular respect to porous oxide systems. In cases when there is no confidence that the whole object is probed by an AC signal, the partial analysis of spectrum fragments is reasonable. Figure 8 demonstrates the fitting of the chosen spectrum fragments.

**Figure 9:**
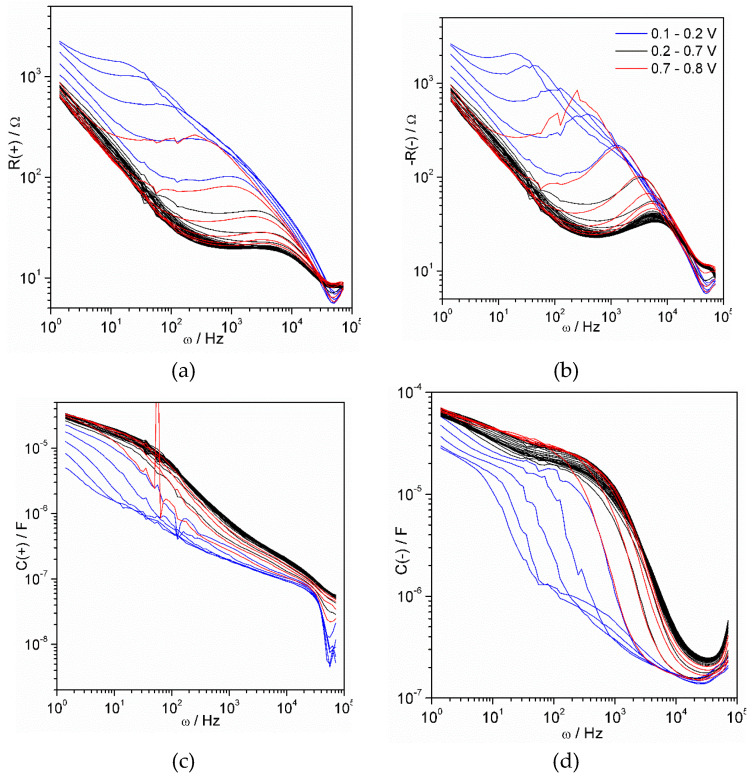
Equivalent circuit parameters estimated for spectrum shown in Figure 8 as functions of alternating current (AC) frequency: R_1_ (**a**), R_2_ (**b**), C_1_ (**c**) and C_2_ (**d**). Sign in brackets designates charge of corresponding electroactive species. The curves corresponding to the left and right borders of the potential scanning range are shown by blue and red colors, respectively.

We have chosen seven-point fragments for partial fitting due to the following reasons. Firstly, the number should be odd so than middle frequency value can be attributed to the calculated parameters. Secondly, the number of points should be about two times bigger than number of sought parameters to avoid intermittent error. The resulting data obtained from analysis of all spectra for the thin (10 cycles) polyaniline film are shown in Figure 9.

All the graphs in Figure 9 contain a set of curves of a similar form. The curves corresponding to potential range between 0.3 V and 0.6 V, where film demonstrates the highest conductivity, are gathered. The most noticeable changes occur within the ranges 0.1–0.3 V and 0.6–0.8 V, when the conductivity of the film increases or decreases. We have found that the least noisy and most reproducible data are obtained in the case of the thinnest film. The data obtained for polyaniline film deposited by 100 cycles were not even able to be treated, whereas data obtained for 30 and 50 cycle-deposited films were extremely noisy. A mathematical analysis described above was successful only in the case of the thinnest, 10 cycle-deposited film. The data were treated according to the theoretical methodology described above.

**Figure 10:**
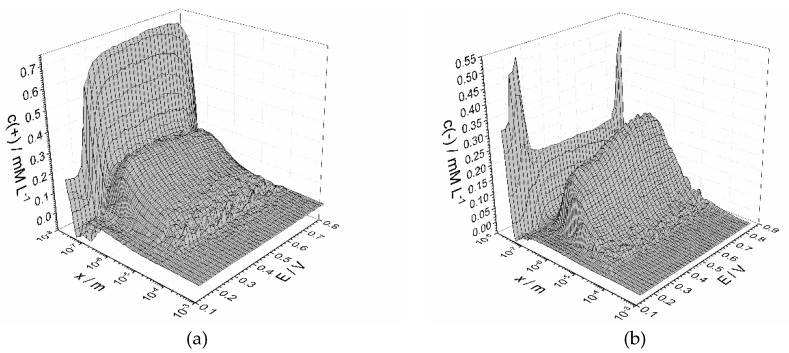
Three-dimensional profiles of distribution of cations (**a**) and anions (**b**) along the distance from electrode surface within the potential range from 0.1 to 0.8 V. Results correspond to polyaniline film obtained by 10 cycles of electropolymerization.

**Figure A3:**
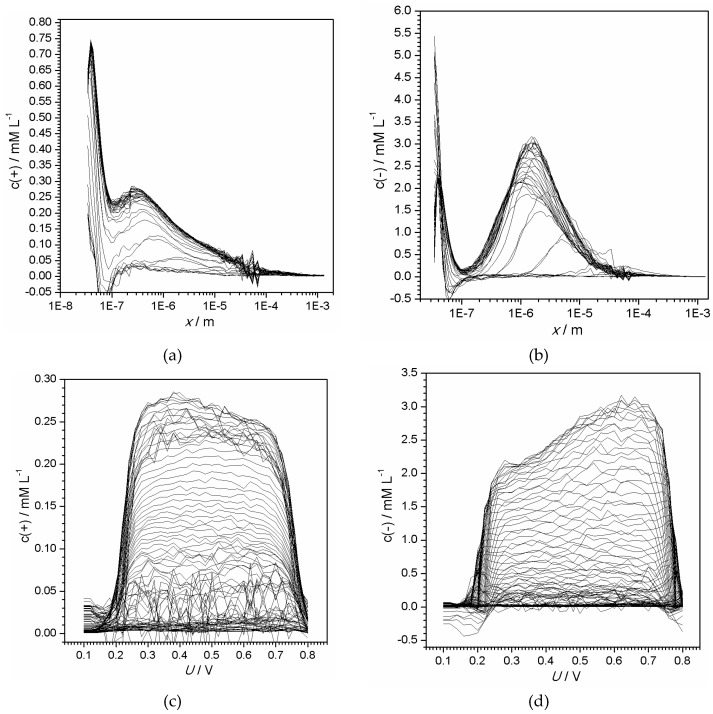
Dependencies of concentration of cations (**a**,**c**) and anions (**b**,**d**) on distance (**a**,**b**) and potential (**c**,**d**)—2D projections of 3D plot onto (Figure 10) *c*-*x* and *c*-*E* planes.

Figure 10 shows the obtained data of concentration as a function of electrode potential and distance from the electrode surface. The 2D projections of the plots shown onto the potential and distance scale is given in Figure A3.

Before discussing the graphs, it is important to emphasize that the technique is able to reveal only charge carriers that are affected by oscillating electrode potential. The inert particles (i.e., big molecular ions) cannot be observed. According to a conventional concept, the role of charge carriers in conductive polymers is played not only by ions but also by polarons. Based on experimental results, only two types of charge carriers of the opposite sign could be detected. They were attributed to absorbed cations and anions from the solution. The impossibility of detection of polarons could be explained by presumed high mobility, suggesting that they move as fast as electric field changes and much higher frequency is required to observe them. Another explanation is based on the eventual incorporation of polaron response in the response of positively charged species—incredibly high measurement precision is required to discriminate between subtle responses of different particles.

The observed concentrations are stipulated by two factors: the influence of electrode polarization and penetration of the ions from solution. Both factors change with distance oppositely. The signal from electrode decays with distance. On the other hand, the amount of ions which can be affected by the AC signal increases towards polymer-solution interface, the latter playing a role of inexhaustible source of ions. Contradiction between two factors explains minima and maxima on the plots in Figure 10. The high values observed at small distances is explained by vicinity to the electrode surface. The following decrease of concentration changes into growth and achieves maximum at about 1 µm distance followed by downfall towards the end of the polymer film. Undoubtedly, the polymer-solution interface cannot be considered as a uniform plane. Macromolecules cannot be expected to have equal lengths and be oriented in the same manner. Therefore, polymer film thickness is not a constant, but a tentative parameter. The apparent negative values were obtained in the borders of potential range at high frequencies of probing signal. The reason for that could be foreseen non-applicability of the used technique at high and low potentials, when electrochemical processes may occur.

A further issue is concerned with potential dependences of estimated values. It is clearly seen that all the values supporting efficient charge transfer have maximal values within the range of 0.3–0.7 V, where polyaniline behaves as a conductor. Exceeding this range leads to loss of conductivity properties followed by reduce of concentration of mobile charge carriers. Although a significant difference of concentrations on potential scale might be expected, all the values remain quite close within the conductivity range. Nevertheless, a slight tendencies deserve paying attention. Concentration of positive ions coefficients is high at low potential and falls with a potential increase (Figure 10a and Figure A3c). The concentration of the ions of negative charge changes in an opposite manner—it grows with a potential increase (Figure 10b and Figure A3d). At relatively low potential conductive polyaniline film contains many cations, mainly represented by hydrogen or hydroxonium ions, which were formed during oxidation. With the potential increase, cations migrate towards the solution and are replaced by incoming anions, that migrate towards electrode due to positive charge progressively formed in an oxidized film.

**Figure 11:**
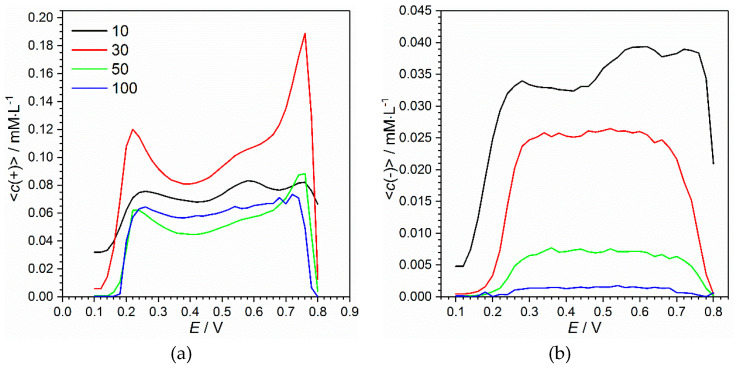
Average concentrations of positively (**a**) and negatively (**b**) charged ions as functions of electrode potential.

The graphs in Figure 10 make up the main set of output data of the impedance spectroscopy-based technique. Although the 2D sections of the graph (Figure A3) are easier for representation of particular tendencies, the 3D mode facilitates recognition and detection of effects that deserve special attention. Generally, we admit that the analysis on a distance scale is not precise. We have conducted the analysis to prospect into further applications of the technique where resolution on a geometric scale would be of high importance. Anyway, one can use the demonstrated approach to evaluate average concentration in the whole film as a function of potential (Figure 11).

A first look at Figure 11 reveals the effect of thickness on the concentration of electroactive species, which is clearly seen in the case of negatively charged ions. The thicker the film is, the less charge carriers it contains. Such a tendency is not so noticeable in the case of cations (Figure 11a). Moreover, average concentration of positive ions decreases not more than by half comparing 10 and 100 cycles, whereas anion concentration drops down intensively with thickening of the film. The phenomenon could be explained based on the nature of the considered ions. The cations are represented by hydroxonium ions (H_3_O^+^) or protons (H^+^) bounded with nitrogen atoms, e.g., in emeraldine form (Appendix A). Taking into account their relatively small size and high mobility in water solution provided by Grotthuss mechanism (proton jumping), their ability to penetrate the film is not affected significantly by the film thickness. The positive ions, in particular, protons, are also generated inside the film during its oxidation and transformation between polyaniline forms. This effect could be a reason for observed high concentration at the border potentials of the conductivity region, which is most noticeable in the case of the film deposited by 30 cycles (Figure 11a). The role of negatively charged anions (Figure 11b) must be played by perchlorate ions (${\mathrm{ClO}}_{4}^{-}$). Their absorption is hindered due to their relatively large size. Therefore, the thickest film contained the least concentration of the anions. No remarkable dependence of concentration on potential within the conductivity region was observed.

## 6. Conclusions

A set of polyaniline films was studied with particular attention on the nature of processes occurring during film polarization. A role of mobile ions localized inside the film has been confirmed to be crucial for polymer conductivity properties. The charge carriers’ behavior was studied by voltammetric, microgravimetric and multiparametric conductivity techniques. For the first time, values of concentration of positive and negative ions have been estimated. An additional extension of the method included analysis in respect of AC signal frequency, which allowed characterization of ion distribution along the polymer film thickness.

## Figures and Tables

**Table 1:** Set of data on number of electropolymerization cycles, corresponding oxidation charge and estimated thickness for chosen samples of polyaniline films.

Cycle Number	Charge Consumed for Electropolymerization/C	Estimated Thickness/m
10	4.212 × 10^−4^	2.6 × 10^−7^
30	3.030 × 10^−3^	1.9 × 10^−6^
50	1.036 × 10^−2^	6.4 × 10^−6^
100	1.034 × 10^−2^	6.4 × 10^−5^

**Scheme A1:**
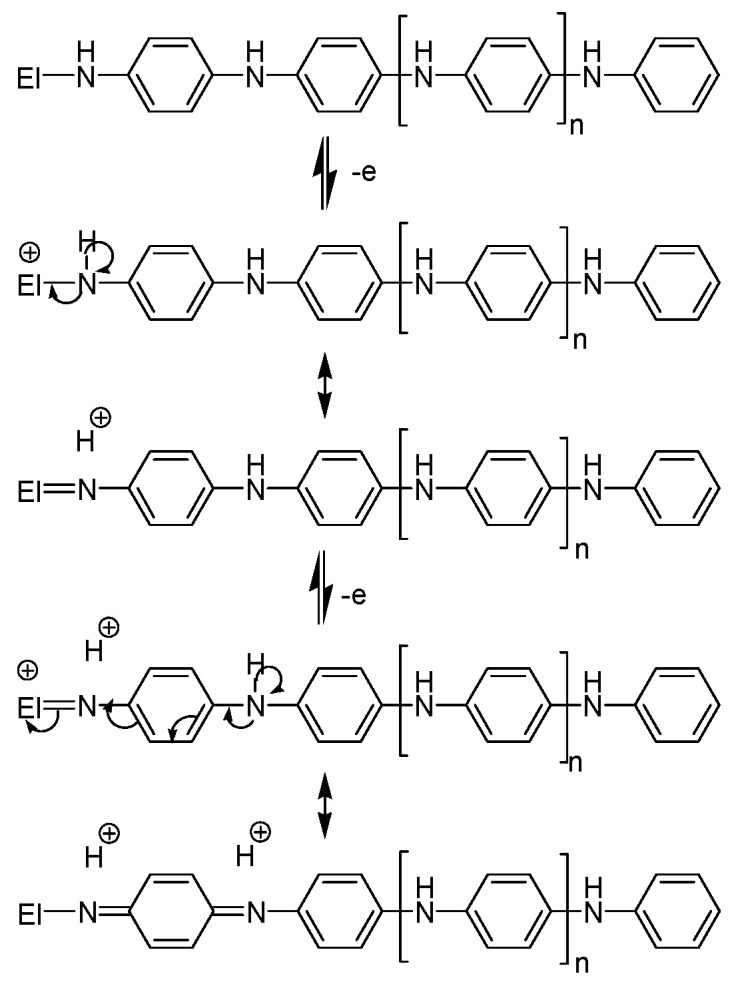
Delivery of charge from electrode onto polyaniline macromolecule forming an oxidized (chinoidal) fragment.

**Scheme A2:**
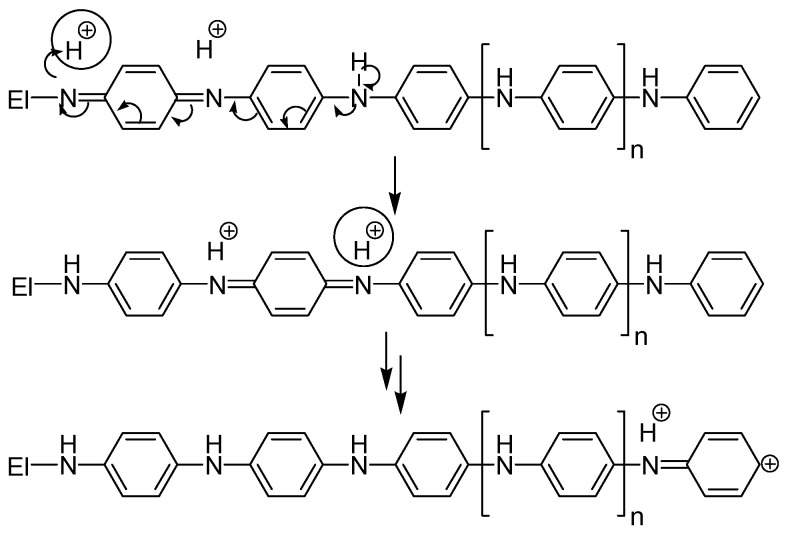
Positive charge transfer along the polyaniline molecule.

**Scheme A3:**
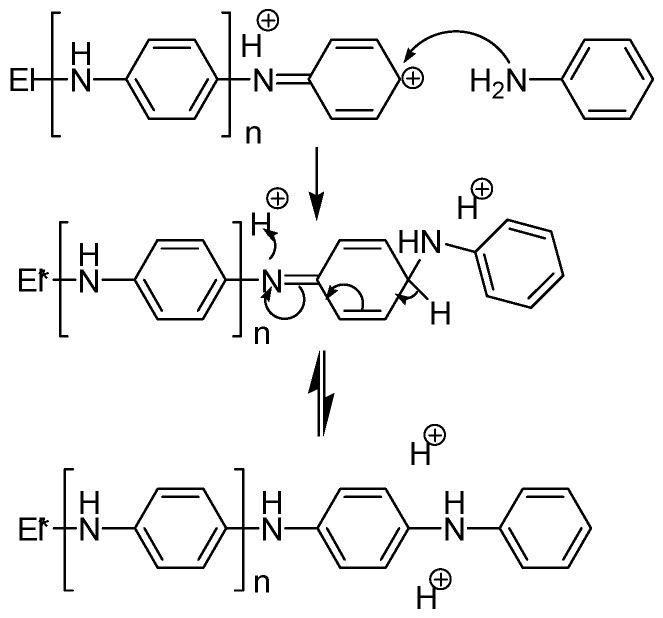
Mechanism of monomer unit addition to the end of the polymer chain.

**Scheme A4:**
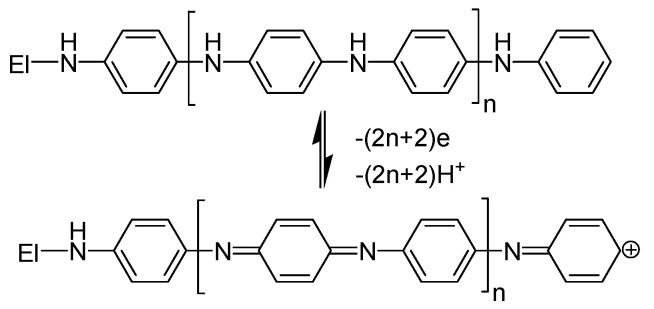
Utmost oxidation of polyaniline causes formation of the pernigraniline structure with alternate oxidized/reduced groups.

## Supplementary Materials

The following are available online at https://www.mdpi.com/1996-1944/13/12/2877/s1. Figure S1, schematic representation of three neighbor discrete layers and difference of potential and concentration of charged species between them. Figure S2, equilibrium profiles of potential, electric field and concentration of charged species for positive and negative electrode potential. Figure S3, illustration of mathematical approach that involves analysis of infinitesimal layer and consequent summation (integration) along the whole film thickness. Figure S4, damping of the probing harmonic signal illustrated as charge oscillation along the distance from the electrode. Figure S5, illustration of current oscillation as a function of *x* and *t*. Figure S6, equivalent circuits corresponding to the worked theoretical model: in general case and in the considered work. Figure S7, fragmentary analysis of the experimental data: fitting of the whole spectrum, fitting of the zoomed fragment. Equations (S1–S60).

## Appendix A

The proposed mechanism of polyaniline film formation by electrochemical addition of aniline monomer units with brief comments is given below. The proposed mechanism is based on general chemical principles.

Starting from a non-oxidized polyaniline chain (*leucoemeraldine* form) attached to the electrode surface, two electron transfers to the electrode causes transformation of a phenylene unit to a chinoidal unit and release of two ions (H^+^ or H_3_O^+^) (Scheme A1). “El” designates electrode surface.

The cations formed are not expected to diffuse freely in the medium between polymer chains, due to interaction with nitrogen atoms and steric obstacles. However, their apparent mobility could be rather high due to an effect similar to the Grotthus mechanism that explains very high mobility of hydroxonium ion. The conjugated structure allows an oxidized fragment to shift along the chain according to Scheme A2.

The form containing one or several chinoidal fragments (called the emeraldine form) is electrically conductive. Scheme A2 illustrates how the positive charge can travel along the chain until the outermost position at the end of the chain. Theoretically, the form is capable of propagation by adding a monomer unit coming from solution. Attaching of the monomer (Scheme A3) harvests the charge of the polymer and reverts it to the initial reduced state. However, at moderately low potential the amount of cations is too small and their migration to the chain end is too slow, to allow stable growth of polymer film.

Increase of electrode potential accelerates the process, but at the same time leads to formation of an utmost oxidized form (*pernigraniline*) which is no longer able to transfer charges (Scheme A4).

In fact, the processes shown are also complicated by the anions which intercalate into polyaniline film due to diffusion and migration caused by the electric field. The objective optimization of the polymer film growth would require its reduction to the initial leucoemeraldine state followed by fast oxidation and subsequent repetition of the cyclic procedure. The cyclic electropolymerization has been proven as an efficient technique for obtaining of a conductive polymer film from monomer solution on the electrode surface.

## Appendix B

Appendix B includes raw measurement and high informative graphical data obtained from processing results.
