# Supplementary material for: An Insight into Ionic Conductivity of Polyaniline Thin Films
Source: Materials (Basel). 2020 Jun 26;13(12):2877. doi: 10.3390/ma13122877 (PMC7344909; doi:10.3390/ma13122877)
Supplement: Supplementary file 1 [file materials-13-02877-s001.pdf]

materials

Supporting information

An Insight into Ionic Conductivity of Polyaniline Thin Films

Pavel Chulkin [1], Mieczysław Łapkowski [1,2,*]

Theoretical Part

The supporting information is dedicated to substantiation of the theoretical basis for analysis of electrochemical impedance spectra. It is worth pointing out that creation of a reasoned mathematical instrument for data treatment makes the most important and time-consuming part of the fundamental physicochemical work. The text represents a single-step mathematical procedure that leads to discovery of the final formula for impedance of the conductive polymer film. The strategy demonstrated was achieved after successive upgrading of the model until satisfactory correspondence with the experimental data. The designations of mathematical symbols and related physicochemical values with appropriate dimensions are enlisted below. In order to facilitate a reader's understanding of the mathematical operations being accomplished to create the physical model, and the procedure is divided in several successive steps.

Constants:

F—Faraday constant (96485 $C\cdot mol^{-1}$);

j—imaginary unit ($\sqrt{-1}$);

ε_0—vacuum permittivity ($8.85\cdot 10^{-12}$ $F\cdot m^{-1}$);

ε—relative material permittivity (–);

R—ideal gas constant (8.314 $J\cdot mol^{-1}\cdot K^{-1}$)

T—temperature (accepted to be constantly equal 298 K)

f—secondary constant, equal to F/RT, introduced for simplification of expressions

Electrochemical parameters:

x—distance (m);

δx—thickness of discrete thin layer (m);

c—concentration ($mol\cdot m^{-3}$);

δc—difference of concentration in two neighbour discrete layers ($mol\ m^{-3}$);

D—diffusion coefficient ($m^2\cdot s^{-1}$);

J—flux ($mol\cdot m^{-2}\cdot s^{-1}$)

Electrical parameters:

i—current density ($A\cdot m^{-2}$);

U—voltage (V);

δU—voltage drop in discrete thin layer (V);

E—electric field intensity ($V\cdot m^{-1}$);

Z, Z_{re}, Z_{im}—impedance (complex value), real and imaginary part (Ω);

Y—admittance (Ω^{-1});

C—capacitance normalised by surface area ($F\ m^{-2}$);

R—resistance normalised by surface area ($\Omega\ m^2$), not confuse with molar Boltsmann constant which is always accompanied by T;

L—inductance ($\Omega\ m^2$).

Time-dependent oscillating values (dimensions are the same as for corresponding stationary values):

Δi—periodic current density oscillation;

ΔU—periodic voltage oscillation;

Δc—periodic concentration oscillation.

Time-independent complex phasors:

$\tilde{i}$ —current phasor;

$\tilde{U}$ —voltage phasor;

$\tilde{c}$—concentration phasor.

1. Current Oscillation under Stationary Conditions

The impedance will be firstly calculated for the one discrete layer containing one type of charge carriers. The obtained formula will then be generalised onto the whole complicated system. A model depicting a single-type charge transfer through a discrete layer is presented in Figure 1. It includes the designations of parameters that will be used in computations hereafter. The direction of current (move of positive charges) is also specified in the figure. It corresponds to the situation when the electrode is on the left side, provided that anodic current is positive.

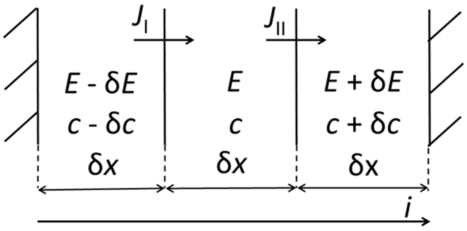

Figure S1. Schematic representation of three neighbour discrete layers and difference of potential and concentration of charged species between them.

The description of conductive polymer film required consideration of the presence of the potential gradient along the film. For many electrochemical systems discussed in the literature the potential gradient has been taken into account only in the double electric layer region. It's effect was restricted to the region between the electrode surface and outer Helmholtz plane, which comprises the ions that are closest to the electrode surface, but are not specifically adsorbed. The electric field is usually considered to have no influence on the delivery of charge carriers beyond the outer Helmholtz plane. The disregard of potential gradient is not reasonable in the case of polymer film. This factor was taken into account in the latest works on conductive polymer impedance study by Láng et al. [1,2].

Since we consider a thin layer, randomly taken from the film, there is no information, available in advance, about potentials on the borders. Therefore, the voltage drop δU within the layer is introduced as a parameter responsible for driving the charge oscillation. It is made to avoid confusion with signs of potentials in case where no reference is used. In the case considered, the increase of absolute value of voltage would always cause an increase of current.

To derive the final formula one has to: (i) consider oscillating parameters affecting current; (ii) represent current oscillation as a function of the other oscillating parameters; (iii) express all the parameters in terms of oscillating current and voltage; and (iv) rearrange the final formula using relation $Z = \tilde{U}/\tilde{i}$.

The flux of charged species under *DC* conditions is described by equation (S1).

$$J = -D\frac{\mathrm{d}c}{\mathrm{d}x} + D\frac{zF}{RT}Ec + \frac{\varepsilon\varepsilon_0}{zF}\frac{\mathrm{d}E}{\mathrm{d}t} \tag{S1}$$

Electric current density (S2) is then proportional to the charge carriers flux (1) multiplied by *zF*.

$$i = -zFD\frac{\mathrm{d}c}{\mathrm{d}x} + z^2 fDFEc + \varepsilon\varepsilon_0\frac{\mathrm{d}E}{\mathrm{d}t} \tag{S2}$$

A discrete form of (S2) contains the ratio $\delta c/\delta x$ instead of the derivative. Thus equation (S3) describes a current passing through a thin layer with thickness δx.

$$i = -zFD\frac{\delta c}{\delta x} + z^2 fDFEc + \varepsilon\varepsilon_0\frac{\mathrm{d}E}{\mathrm{d}t} \tag{S3}$$

The equations delivered above will be useful for the estimation of oscillating values. One has to remember that the film was characterised under stationary conditions and relatively low polarisation in order to prevent degradation or progressing doping of the polymer. The stationary current was equal to zero (S4) during impedance registration. That condition is useful to relate stationary concentration gradient with the electric field (S5).

$$-zFD\frac{\mathrm{d}c}{\mathrm{d}x} + z^2 fFDEc = 0 \tag{S4}$$

$$\frac{\mathrm{d}c}{\mathrm{d}x} = zfEc \tag{S5}$$

One could notice that in case of constant electric field along the film thickness (S6):

$$\frac{\mathrm{d}E}{\mathrm{d}x} \approx 0 \tag{S6}$$

the solution of equation (S5) is presented by formula (S7a) or (S7b):

$$c = c_0 \cdot e^{zfEx} \tag{S7a}$$

$$c = c_\mathrm{d} \cdot e^{zfE(x-d)} \tag{S7b}$$

where c_0 is concentration at $x = 0$ and c_d is the concentration at distance d from electrode surface. The decision on using (S7a) or (S7b) is made based on available data. In this work, we will refer the results to the concentration at the outer border of polymer film (film–solution interface), thus (S7b) would be preferable.

The concentration plots are defined by the sign of the species charge (z value). Since z value will become crucial in determination of ion type, one has to be careful with the z coefficient during all computation procedures. The schematic Figure S2 based on the Gouy–Chapman model is suggested to be used as a reference for qualitative check of the correctness of the formulas.

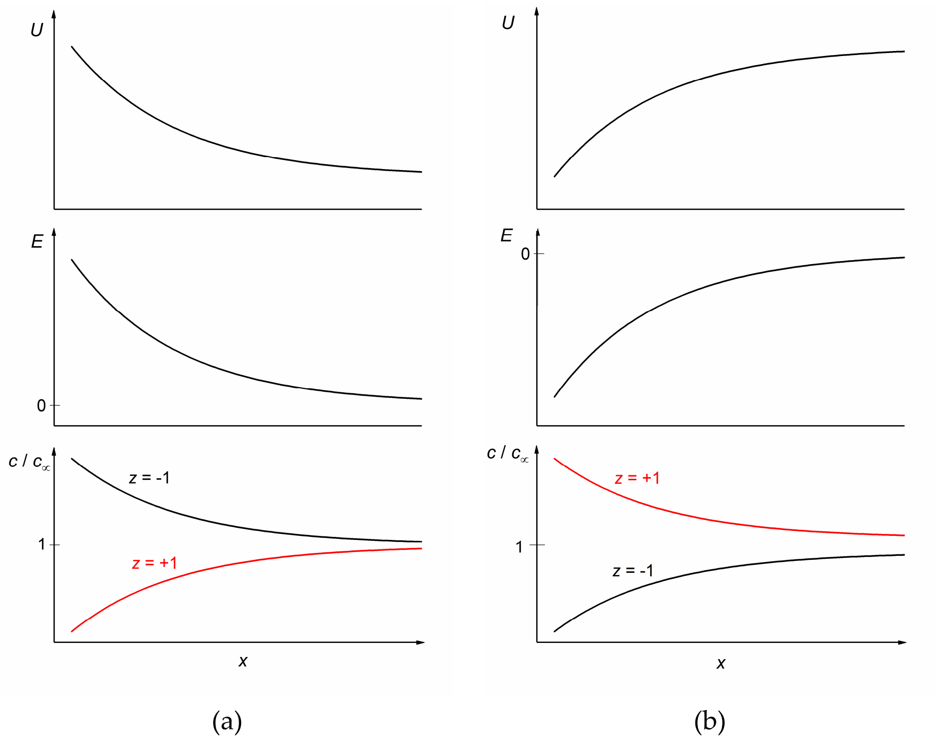

Figure S2. Equilibrium profiles of potential, electric field and concentration of charged species for positive (**a**) and negative (**b**) electrode potential.

According to the Poisson equation, the condition (S6) is reasonable if the space charge of the film is close to zero. The expectation may seem presumptuous, so we will not base our computations on it and consider it only in respect of final formula.

2. Oscillation of the Concentration of the Charged Species

The next main step is concerned with phasor $\tilde{c}$ that has to be expressed in terms of current and voltage. For determining oscillation of concentrations in middle layer depicted in Figure S1 one has to consider oscillation of fluxes J_{I} and J_{II} (S8 a, b). The formulas were obtained by adding or subtracting δ terms in Equation (S1).

$$\Delta J_{\mathrm{I}} = -D\frac{1}{\delta x}\Delta c + zfD\left(c-\delta c\right)\Delta E + zfD\left(E-\delta E\right)\Delta c + \frac{\varepsilon\varepsilon_0}{zF}\frac{\mathrm{d}\Delta E}{\mathrm{d}t} \tag{S8a}$$

$$\Delta J_{\mathrm{II}} = -D\frac{1}{\delta x}\Delta c + zfDc\Delta E + zfDE\Delta c + \frac{\varepsilon\varepsilon_0}{zF}\frac{\mathrm{d}\Delta E}{\mathrm{d}t} \tag{S8b}$$

Oscillation of concentration must obey Fick's second law (S9):

$$\frac{\partial \Delta c}{\partial t} = -\frac{\partial \Delta J}{\partial x} \tag{S9}$$

transformed into discrete form (S10):

$$\frac{\partial \Delta c}{\partial t} = \frac{\Delta J_{\mathrm{I}} - \Delta J_{\mathrm{II}}}{\delta x} \tag{S10}$$

Expansion of (S10) using (S8a,b) and introduction of phasors instead of Δ-values leads to (S11):

$$\frac{\partial \Delta c}{\partial t} = -\frac{zfD}{\delta x}\left(\Delta E\cdot\delta c + \Delta c\cdot\delta E\right)$$

$$\downarrow$$

$$\frac{\partial \tilde{c}\exp\left(j\omega t\right)}{\partial t} = -\frac{zfD}{\delta x}\left(\tilde{E}\exp\left(j\omega t\right)\cdot\delta c + \tilde{c}\exp\left(j\omega t\right)\cdot\delta E\right)$$

$$\downarrow$$

$$j\omega\frac{\partial \tilde{c}}{\partial t}\exp\left(j\omega t\right) = -\frac{zfD}{\delta x}\left(\tilde{E}\cdot\delta c + \tilde{c}\cdot\delta E\right)\exp\left(j\omega t\right)$$

$$\downarrow$$

$$j\omega\tilde{c} = -\frac{zfD}{\delta x}\left(\tilde{E}\cdot\delta c + \tilde{c}\cdot\delta E\right) \quad \text{(S11)}$$

$$\downarrow$$

$$\tilde{c} = -zfD\frac{\mathrm{d}c}{\mathrm{d}x}\frac{1}{j\omega + zfD\dfrac{\mathrm{d}E}{\mathrm{d}x}}\cdot\tilde{E} \quad \text{(S12a)}$$

$$\text{or,}\ \tilde{E} = -\frac{\left(j\omega + zfD\dfrac{\mathrm{d}E}{\mathrm{d}x}\right)}{zfD\dfrac{\mathrm{d}c}{\mathrm{d}x}}\cdot\tilde{c} \quad \text{(S12b)}$$

It is worth noting that according to (S12) the phase shift between electric field and concentration oscillation depends on the ratio between frequency and electric field gradient (two terms in denominator of (S15a) and numerator of (S15b)). When $zfD\cdot\mathrm{d}E/\mathrm{d}x \ll \omega$ or when condition (S6) is obeyed, the concentration and electric field oscillate in antiphase. In the opposite case ($zfD\cdot\mathrm{d}E/\mathrm{d}x \gg \omega$) both values oscillate in-phase, like charge and voltage of an ideal capacitor. The first case will be considered in this work, since it was proven to be in agreement with experimental results. However, the denoted relationship between complex electric field and concentration of charge carriers deserves further development to obtain a universal model of charge transport under alternating current (AC) conditions.

3. Alternating Current (AC) Response of the Model Thin Layer

The current oscillation is obtained based on formula (S9) taking into account zF coefficient between electrical current and flux of charged species (S13).

$$zF\frac{\partial \Delta c}{\partial t} = -\frac{\partial \Delta i}{\partial x} \quad \text{(S13)}$$

Following set of rearrangements aimed at replacement Δ-terms by phasors in much the same way as shown in (S11), one gets:

$$\frac{\partial \tilde{i}}{\partial x} = -j\omega zF\tilde{c} \quad \text{(S14)}$$

The discrete form of (S14) is

$$\tilde{i} = -j\omega zF\tilde{c}\cdot\delta x \quad \text{(S15)}$$

The expression for $\tilde{c}$ is imported from (S13) and the equation is consequently rearranged:

$$\tilde{i} = j\omega zF\delta x \cdot zfD\frac{\mathrm{d}c}{\mathrm{d}x}\frac{1}{j\omega + zfD\dfrac{\mathrm{d}E}{\mathrm{d}x}} \cdot \frac{\delta\tilde{U}}{\delta x}$$

$$\downarrow$$

$$\delta\tilde{U} = \frac{j\omega + zfD\dfrac{\mathrm{d}E}{\mathrm{d}x}}{j\omega z^2 fFD\dfrac{\mathrm{d}c}{\mathrm{d}x}}\tilde{i}$$

$$\downarrow$$

$$\delta\tilde{U} = \left(\frac{1}{z^2 fFD\dfrac{\mathrm{d}c}{\mathrm{d}x}} + \frac{\dfrac{\mathrm{d}E}{\mathrm{d}x}}{j\omega zF\dfrac{\mathrm{d}c}{\mathrm{d}x}}\right)\tilde{i} \tag{S16}$$

On the last stage it was managed to split the expression in two parts that represent different AC frequency dependences. One could already perceive that the first term corresponds to resistive behaviour (no frequency dependence) whereas the second one stands for capacitive properties (see Table 2 for impedance of basic physical elements).

The formula represents a relationship between voltage and current in an infinitesimal layer (Figure S3). The impedance of the whole film can be calculated by integrating (S16).

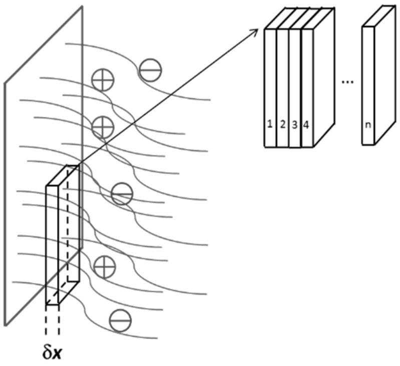

Figure S3. Illustration of mathematical approach that involves analysis of infinitesimal layer and consequent summation (integration) along the whole film thickness.

One would think that the impedance of the whole film of thickness *d* could be obtained by direct integration of (S16) by *x* from 0 to *d*. However, such an apparently obvious approach would still be inchoate. The set of rearrangements (S17) demonstrates that direct integration of impedance (dZ) would only be reasonable if current oscillation is constant along the *x* coordinate.

$$\tilde{U} = \int_d \mathrm{d}\tilde{U}(x) = \int_d \left(Z(x)\cdot\tilde{i}\right) = \left[\text{if } \tilde{i} \neq f(x)\right] = \tilde{i}\cdot\int_d \left(Z(x)\right) = \tilde{i}\cdot Z_{\text{total}} \tag{S17}$$

In the real system, such a condition is not expected to be held a priori. A justification is required to either prove or disprove the statement. The effect of damping of current oscillation along the *x* scale was called into question by authors during development of the theory. In all cases, neglecting the current damping caused complete disagreement between model and experimental data. In order to take into account current oscillation decay with the distance from the electrode–film interface (as shown in Figure S4a), one has to introduce a damping coefficient $\gamma(x)$ and use it in the formula $i(x) = i_0 \cdot \gamma(x)$, where i_0 is current amplitude on the electrode–film interface, i.e., directly related to the Δi value registered by the measurement equipment:

$$\tilde{U} = \int_0^d \frac{\mathrm{d}Z(x)}{\mathrm{d}x} \cdot \tilde{i}_0 \cdot \gamma(x) \cdot \mathrm{d}x \quad \text{(S18)}$$

and the impedance of the whole system is calculated as:

$$Z = \int_0^d \frac{\mathrm{d}Z(x)}{\mathrm{d}x} \cdot \gamma(x) \cdot \mathrm{d}x \quad \text{(S19)}$$

The first factor in (S19), i.e. impedance increment, has been already obtained above (S16). The following two sections will be dedicated to obtaining the analytical form of the $\gamma(x)$ term.

4. Damped Oscillation of the Ion Concentration under AC Conditions

The illustration in the beginning of the section (Figure S4) is intended to facilitate a reader's understanding of the purpose of the following computations. The effect of the AC signal that fades away along the distance from the electrode is illustrated in Figure S4a. The attenuation of damping depends on signal frequency (Figure S4b). The high-frequency signal would fade away faster and oscillation would not go far from the source (red line in Figure S4b), whereas the low-frequency signal of the same amplitude would reach longer distance (blue line in Figure S4b). Due to this effect, the response of the system would be more complicated than a response of physical equivalent circuit.

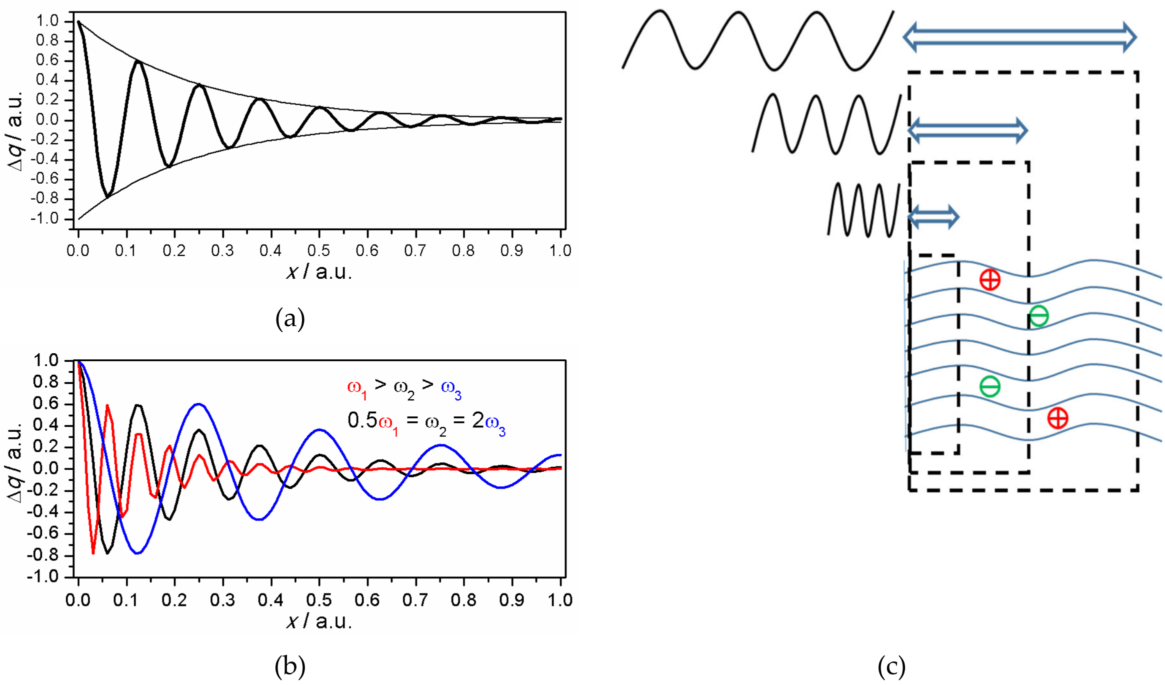

Figure S4. Damping of the probing harmonic signal illustrated as charge oscillation along the distance from the electrode: a—attenuation of the signal amplitude; b—effect of the frequency on signal dumping; *c*—control of the size of the monitored region by change of the probing frequency. The schematic illustration is based on the discussions below.

As a form of primitive visualization, one may consider that the frequency of the signal defines the size of the layer to be covered (Figure S4c). Figure S4c can only be used for a superficial explanation of the phenomenon. In reality, there is no cut-off edge of the wave and the signal attenuates along the whole thickness (Figure S4b).

A functional dependence $\tilde{i} = f(x)$ obtained in (S14) and (S15) included the concentration phasor ($\tilde{c}$). The only way to estimate the concentration phasor is to consider the transport Equation (S1) and

Fick's diffusion law (S9). Equation (S1) is used without the last term. The explanation for its disregard is given in Part 2. Oscillation of the concentration of the charged species. The rearrangements, that were made to proceed from (S35) to (S36), are analogical to those described in Part 2 so they are not commented on here.

$$\Delta J = -D\frac{\partial \Delta c}{\partial x} + zfD\left(E\cdot\Delta c + c\cdot\Delta E\right) \tag{S20}$$

$$\downarrow$$

$$\frac{\partial \Delta c}{\partial t} = -\frac{\partial}{\partial x}\left(-D\frac{\mathrm{d}\Delta c}{\mathrm{d}x} + zfD\left(E\cdot\Delta c + c\cdot\Delta E\right)\right)$$

$$\downarrow$$

$$\frac{\partial \Delta c}{\partial t} = D\frac{\partial^2 \Delta c}{\partial x^2} - zfD\left(E\cdot\frac{\partial \Delta c}{\partial x} + \Delta c\cdot\frac{\partial E}{\partial x} + c\cdot\frac{\partial \Delta E}{\partial x} + \Delta E\cdot\frac{\partial c}{\partial x}\right)$$

$$\downarrow$$

$$\boxed{j\omega\tilde{c} = D\frac{\partial^2 \tilde{c}}{\partial x^2} - zfD\left(E\cdot\frac{\partial \tilde{c}}{\partial x} + \tilde{c}\cdot\frac{\partial E}{\partial x} + c\cdot\frac{\partial \tilde{E}}{\partial x} + \tilde{E}\cdot\frac{\partial c}{\partial x}\right)} \tag{S21}$$

The last form represents the whole set of parameters that affect concentration oscillation. However, its further analytical treatment is not possible. For that reason, here we will present the solution based on minimal approximations that finally allowed us to achieve the satisfactory accordance between the theory and experimental data.

Here, we will demonstrate a solution which is reasonable, if condition (S6) is accepted. The third term, including electric field gradient, is cancelled and the fifth term, including concentration gradient, is modified according to (S7). Thus the equation is rearranged to (S22).

$$j\omega\tilde{c} = D\frac{\partial^2 \tilde{c}}{\partial x^2} - zfD\left(E\cdot\frac{\partial \tilde{c}}{\partial x} + c\cdot\frac{\partial \tilde{E}}{\partial x} + \tilde{E}\cdot zfEc_0\cdot e^{zfEx}\right) \tag{S22}$$

Now, Equation (S20b) should be addressed to obtain the final differential equation in respect to the $\tilde{c}\left(x\right)$ function. Condition (S12b) is simplified to (S24) by taking into account (S6) and (S7).

$$\tilde{E} = -\frac{\left(j\omega + zfD\frac{\mathrm{d}E}{\mathrm{d}x}\right)}{zfD\frac{\mathrm{d}c}{\mathrm{d}x}}\cdot\tilde{c}$$

$$\downarrow$$

$$\tilde{E} = -\frac{j\omega\tilde{c}}{zfD\frac{\mathrm{d}c}{\mathrm{d}x}}$$

$$\downarrow$$

$$\tilde{E} = -\frac{j\omega\tilde{c}}{z^2f^2DEc_0}\cdot e^{-zfEx} \tag{S23}$$

$$\tilde{E} = A\tilde{c} \tag{S24}$$

where A is a x-dependent coefficient relating phasors of E and c. In the case, considered here, A is an imaginary negative value exponentially decreasing or increasing with x. Additionally, the later

discussions are expected to involve x-independent part of A, which we suggest designating by letter B to avoid confusion with manifold parameters in the equations.

$$A = -\frac{j\omega}{z^2 f^2 D E c_0} \cdot e^{-zfEx} \tag{S25}$$

$$B = -\frac{j\omega}{z^2 f^2 D E c_0} \tag{S26}$$

The last preparation, before construction of the final equation, is expression of the E-phasor derivative, which is also involved in (S22).

$$\frac{\mathrm{d}\tilde{E}}{\mathrm{d}x} = \frac{\mathrm{d}}{\mathrm{d}x}\left(-\frac{j\omega\tilde{c}}{z^2 f^2 D E c_0} \cdot e^{-zfEx} \right) = -\frac{j\omega}{z^2 f^2 D E c_0} \frac{\mathrm{d}}{\mathrm{d}x}\left(\tilde{c} \cdot e^{-zfEx} \right) =$$

$$= -\frac{j\omega}{z^2 f^2 D E c_0} \frac{\mathrm{d}}{\mathrm{d}x}\left(\tilde{c} \cdot e^{-zfEx} \right) = -\frac{j\omega}{z^2 f^2 D E c_0} \left(\frac{\mathrm{d}\tilde{c}}{\mathrm{d}x} \cdot e^{-zfEx} - zfE\tilde{c} \cdot e^{-zfEx} \right) =$$

$$= -\frac{j\omega}{z^2 f^2 D E c_0} e^{-zfEx} \left(\frac{\mathrm{d}\tilde{c}}{\mathrm{d}x} - zfE\tilde{c} \right) = A\left(\frac{\mathrm{d}\tilde{c}}{\mathrm{d}x} - zfE\tilde{c} \right) \tag{S27}$$

The derivative has been calculated in advance in order to facilitate notation and solution of the equation (S22). Then, by substitutions of $c(x)$ by function (S7) and just obtained $\mathrm{d}\tilde{E}/\mathrm{d}x$ (S27), one obtains the differential Equation (S28).

$$j\omega\tilde{c} = D\frac{\partial^2 \tilde{c}}{\partial x^2} - zfD\left(E \cdot \frac{\partial \tilde{c}}{\partial x} + c_0 \cdot e^{zfEx} \times A\left(\frac{\mathrm{d}\tilde{c}}{\mathrm{d}x} - zfE\tilde{c} \right) + A \cdot \tilde{c} \cdot zfEc_0 \cdot e^{zfEx} \right)$$

$$\downarrow$$

$$j\omega\tilde{c} = D\frac{\partial^2 \tilde{c}}{\partial x^2} - zfD\left(E \cdot \frac{\partial \tilde{c}}{\partial x} + c_0 \cdot B\left(\frac{\mathrm{d}\tilde{c}}{\mathrm{d}x} - zfE\tilde{c} \right) + B \cdot \tilde{c} \cdot zfEc_0 \right) \tag{S28}$$

After simplification and separation of derivatives by the degrees one obtains:

$$D\frac{\mathrm{d}^2 \tilde{c}}{\mathrm{d}x^2} - j\omega\tilde{c} - zfDE\frac{\mathrm{d}\tilde{c}}{\mathrm{d}x} - zfDBc_0\frac{\mathrm{d}\tilde{c}}{\mathrm{d}x} + z^2 f^2 DBEc_0\tilde{c} - z^2 f^2 DBEc_0\tilde{c} = 0$$

$$\downarrow$$

$$\boxed{D\frac{\mathrm{d}^2 \tilde{c}}{\mathrm{d}x^2} - zfD\left(E + Bc_0 \right)\frac{\mathrm{d}\tilde{c}}{\mathrm{d}x} - j\omega\tilde{c} = 0} \tag{S29}$$

The equation can be solved by the standard approach for linear differential equation. The solution of the equation has a form of (S30) where λ_1 and λ_2 are the roots of characteristic Equation (S31).

$$\tilde{c}(x) = C_1 e^{\lambda_1 x} + C_2 e^{\lambda_2 x} \tag{S30}$$

$$D\lambda^2 - zfD\left(E + Bc_0 \right)\lambda - j\omega = 0 \tag{S31}$$

$$\lambda_{1,2} = \frac{zf\left(E + Bc_0\right) \pm \sqrt{z^2 f^2 \left(E + Bc_0\right)^2 + 4\,j\omega/D}}{2} \tag{S32}$$

The constants C_1 and C_2 are defined using boundary conditions. Here, we regard the function $c(x)$ in respect to the oscillation at $x = 0$ (S33a). Another boundary condition (S33b) arises from the obvious assumption that the concentration oscillation decays as it moves away from the boundary.

$$\tilde{c}\left(x\right)\Big|_{x=0} = \tilde{c}_0 \tag{S33a}$$

$$\tilde{c}\left(x\right)\Big|_{x\to\infty} = 0 \tag{S33b}$$

It is obvious that roots λ (S47) are of the opposite sign: $\lambda_1 > 0$, $\lambda_2 < 0$. The condition (S33b) requires only negative exponential term to be valid, and that is why C_1 equals zero. The remaining coefficient is equalised due to condition (S33a), thus giving a final formula for the oscillation of concentration (S34).

$$\boxed{\tilde{c}\left(x\right) = \tilde{c}_0 e^{\frac{zf\left(E+Bc_0\right)-\sqrt{z^2 f^2\left(E+Bc_0\right)^2+4\,j\omega/D}}{2}x}} \tag{S34}$$

Bearing in mind complexity of the formula above, it is worth addressing some limiting cases that would be characterised by simpler functional dependence.

I. In a case when $z^2 f^2 \left(E + Bc_0\right)^2 >> 4\,j\omega/D$ the formula (S34) will be transformed to (S35):

$$\tilde{c}\left(x\right) = \tilde{c}_0 \tag{S35}$$

The limiting condition should be analysed to predict, which experimental parameters, namely E and ω, would lead to the situation when concentration oscillation is apparently constant along the distance from the electrode.

$$z^2 f^2 \left(E + Bc_0\right)^2 >> 4\,j\omega/D \tag{S36}$$

$$\downarrow$$

$$z^2 f^2 \left(E - \frac{j\omega}{z^2 f^2 D E c_0} c_0\right)^2 >> 4\,j\omega/D$$

$$\downarrow$$

$$\left| E - \frac{j\omega}{z^2 f^2 D E} \right| >> \frac{2\sqrt{j\omega/D}}{zf}$$

The rooting at the previous step produced the appearance of the z value which would determine the sign. The original condition (S36) included only a positive term z^2. Thus, the z term must be excluded with a view to not abuse the original condition (S36) and not to complicate further consideration of the inequality. Despite the module in the right part of the last inequality, we will consider the case of positive E implying the symmetrical solution for negative E.

For further processing, the right part has to be decomposed into real and imaginary parts taking into account that:

$$\sqrt{j} = \left(1 - j\right)/\sqrt{2} \tag{S37}$$

$$\left(E - \frac{j\omega}{f^2 DE}\right) >> \frac{1}{f}\sqrt{\frac{\omega}{2D}} - j\frac{1}{f}\sqrt{\frac{\omega}{2D}}$$

$$\downarrow$$

$$E >> \frac{1}{f}\sqrt{\frac{\omega}{2D}} \text{ and } \frac{j\omega}{f^2 DE} << j\frac{1}{f}\sqrt{\frac{\omega}{2D}}$$

$$\downarrow$$

$$E >> \frac{1}{f}\sqrt{\frac{\omega}{2D}} \text{ and } E >> \frac{1}{f}\sqrt{\frac{2\omega}{D}} \quad \text{(S38)}$$

processing of both real and imaginary parts leads to the consistent results. We conclude that the concentration oscillation does not depend on the distance in case when either the electric field is very high or the frequency is very small.

II. In case when $z^2 f^2 \left(E + Bc_0\right)^2 << 4\, j\omega/D$ the formula (S34) will be transformed to:

$$\tilde{c}\left(x\right) = \tilde{c}_0 e^{-\sqrt{\frac{j\omega}{D}}x} \quad \text{(S39)}$$

This solution corresponds to the oscillation decay for infinite diffusion, which is well known from the literature. The function (S39) itself usually is not considered, because traditional impedance formulas are concerned with the solution–electrode interface.

The analogical treatment of the boundary condition would result in the conclusion that the formula (S39) is justified, provided that:

$$E << \frac{1}{f}\sqrt{\frac{\omega}{D}} \quad \text{(S40)}$$

For instance, taking the diffusion coefficient of $1 \cdot 10^{-10}$ $m^2 \cdot s^{-1}$ (approximate value for ions in water solution), the lowest frequency of 0.01 Hz and f equal 38.9 V^{-1} (at 298 K) the condition (S40) would require E to be much less than 257 $V \cdot m^{-1}$.

Comparing reasonability of the approximations **I** and **II**, the last one is expected to be more reasonable under standard experimental conditions for polymer film investigation. The small voltage gradient is ensured by conductivity of the film and mobility of the ions that could freely migrate to eliminate growth of the space charge. If one tries to strongly increase or decrease electrode potential, to get condition **I** (S38) instead of **II** (S40), the only result would be the launch of electrochemical process causing irreversible destruction of the polymer film. The high electric fields are achievable in solid state architecture, as e.g., Organic Light Emmiting Diode (OLED) transport layers.

5. The Final Formula for Impedance of Conductive Polymer Film

At the current stage all intermediate computations have been accomplished, so one has to recall the basic formulas from the previous sections. Substituting (S39) into (S14) one gets:

$$j\omega zF\tilde{c}_0 e^{-\sqrt{\frac{j\omega}{D}}x} = -\frac{\partial \tilde{i}}{\partial x} \quad \text{(S41)}$$

which after integration gives a function of current oscillation on distance:

$$\tilde{i}(x)\Big|_{\tilde{i}_0}^{\tilde{i}(x)} = zF\tilde{c}_0\sqrt{j\omega D}e^{-\sqrt{\frac{j\omega}{D}}x}\Bigg|_0^x \tag{S42}$$

When $x = 0$, $\tilde{i} = \tilde{i}_0$, then the final formula is:

$$\boxed{\tilde{i}(x) = \tilde{i}_0 e^{-\sqrt{\frac{j\omega}{D}}x}} \tag{S43}$$

with

$$\tilde{i}_0 = zF\tilde{c}_0\sqrt{j\omega D} \tag{S44}$$

It is important to extract the value of current amplitude i_0 as a function of distance. The exponent term has to be represented as a sum of real and imaginary parts using the relation (S37) for the square root of the imaginary unit and relation for amplitude and complex phasor of the oscillating value:

$$\Delta i(x,t) = i_0 e^{j\omega t} e^{-\sqrt{\frac{\omega}{2D}}x} e^{-j\sqrt{\frac{\omega}{2D}}x} \tag{S45}$$

after separating real and imaginary parts:

$$\Delta i(x,t) = i_0 e^{-\sqrt{\frac{\omega}{2D}}x} e^{j\left(\omega t - \sqrt{\frac{\omega}{2D}}x\right)} \tag{S46}$$

with the imaginary part presented in trigonometric form:

$$\Delta i(x,t) = i_0 e^{-\frac{x}{\chi}}\left(\cos\left(\omega t - \frac{x}{\chi}\right) + j\sin\left(\omega t - \frac{x}{\chi}\right)\right) \tag{S47}$$

The oscillation in x-scale is damping with a characteristic length:

$$\chi = \sqrt{\frac{2D}{\omega}} \tag{S48}$$

the physical sense of χ is similar to that of diffusion length. When x value is increased by χ, the oscillation amplitude decreases e times.

The last function is harmonic in respect to two parameters: time and distance. The oscillation periods in time and distance scales are shown below:

$$T_t = \frac{2\pi}{\omega} \tag{S49a}$$

$$T_x = 2\pi\sqrt{\frac{2D}{\omega}} \tag{S49b}$$

The form (S47) is more convenient to regard harmonic oscillations. Some of the graphic illustrations of the signal intensity are shown in Figure S5: the distance profiles at seven different time moments within the increment of 1/7 of period (T) (Figure S5a) and the time profiles at 10 chosen distances within the increment of 1/5 χ (Figure S5b).

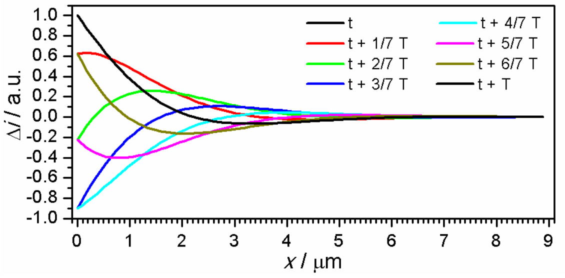

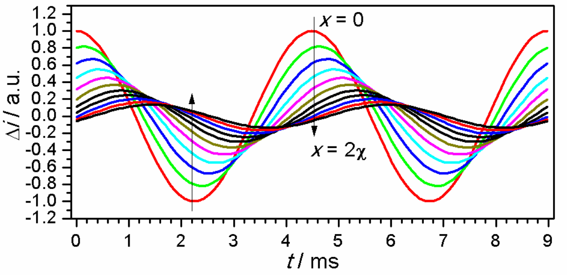

Figure S5. Illustration of current oscillation as a function of *x* (a) and *t* (b). Curves corresponding to different time moments (a) and different distances (b) are brought out by colours. The simulated data correspond to the values: $D = 1\cdot10^{-8}$ m$^2\cdot$s^{-1}, $\omega = 1$ kHz. The vertical arrows indicate signal attenuation with increase of distance.

The exponential term in amplitude (S47) is the sought damping factor $\gamma(x)$ (S18):

$$\gamma(\omega, x) = e^{-\sqrt{\frac{\omega}{2D}}x} \tag{S50}$$

Unfortunately, the formula (S19) cannot be integrated in analytical form because the values and functional dependences $c(x)$, $E(x)$ remain unknown. For that reason, we decided to consider impedance as a sum of impedances of infinitesimal elements (Figure S3):

$$Z = \sum_n \left(\frac{1}{z^2 fFD\frac{\mathrm{d}c}{\mathrm{d}x}} + \frac{1}{j\omega zF\frac{\mathrm{d}c}{\mathrm{d}E}} \right) e^{-\sqrt{\frac{\omega}{2D}}x} \tag{S51}$$

within the thin layer the approximate relationship between concentration and electric field (S7) could be applied to simplify the derivatives.

$$Z = \sum_n \left(\frac{1}{z^3 f^2 FDEc} + \frac{1}{j\omega z^2 fF(\delta x)\cdot c} \right) e^{-\sqrt{\frac{\omega}{2D}}x}$$

$$\downarrow$$

$$Z(\omega) = \sum_n \left(\frac{1}{z^3 f^2 FDE(x)c(x)} + \frac{1}{j\omega z^2 fF(\delta x)\cdot c(x)} \right) \gamma(\omega, x) \tag{S52}$$

The latter formula can be rearranged to the form of impedance (S53) of a physical circuit shown in Figure S6. The z^2 can be eliminated from the formula because its value is always equal to unity:

$$Z = R + \frac{1}{j\omega C} \tag{S53}$$

with

$$R(\omega) = \sum_{i=1}^{n} \frac{1}{zf^2 FDE(x_i)c(x_i)} \gamma(\omega, x_i) \tag{S54}$$

and

$$\frac{1}{C(\omega)} = \sum_{i=1}^{n} \frac{1}{fF(\delta x) \cdot c(x_i)} \gamma(\omega, x_i) \tag{S55}$$

The formula (S53) describes an equivalent electric circuit which comprises a resistor and a capacitor connected in series. In the case of the presence of several charge carriers, their effect on current would be summarised. That means that the model would contain several parallel branches, each corresponding to certain type of ion (Figure S6a). During the analysis of experimental data it was found that two branches (Figure S6b) are enough to fit the spectrum. Of course, adding of additional branches would only improve the fitting, but there was no evidence of necessity of those additional parameters.

The fact that the parameter values are dependent on frequency may be confusing and non-consistent with the conventional concept of equivalent circuits. To explain this feature, we state that the parameters are estimated experimentally in a very narrow range of frequencies, so that ω value in the formulas could be accepted to be constant (Figure S7).

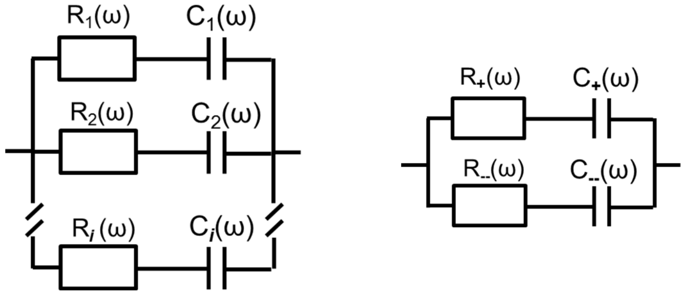

(a) (b)

Figure S6. Equivalent circuits corresponding to the worked theoretical model: in general case (**a**) and in the considered work (**b**).

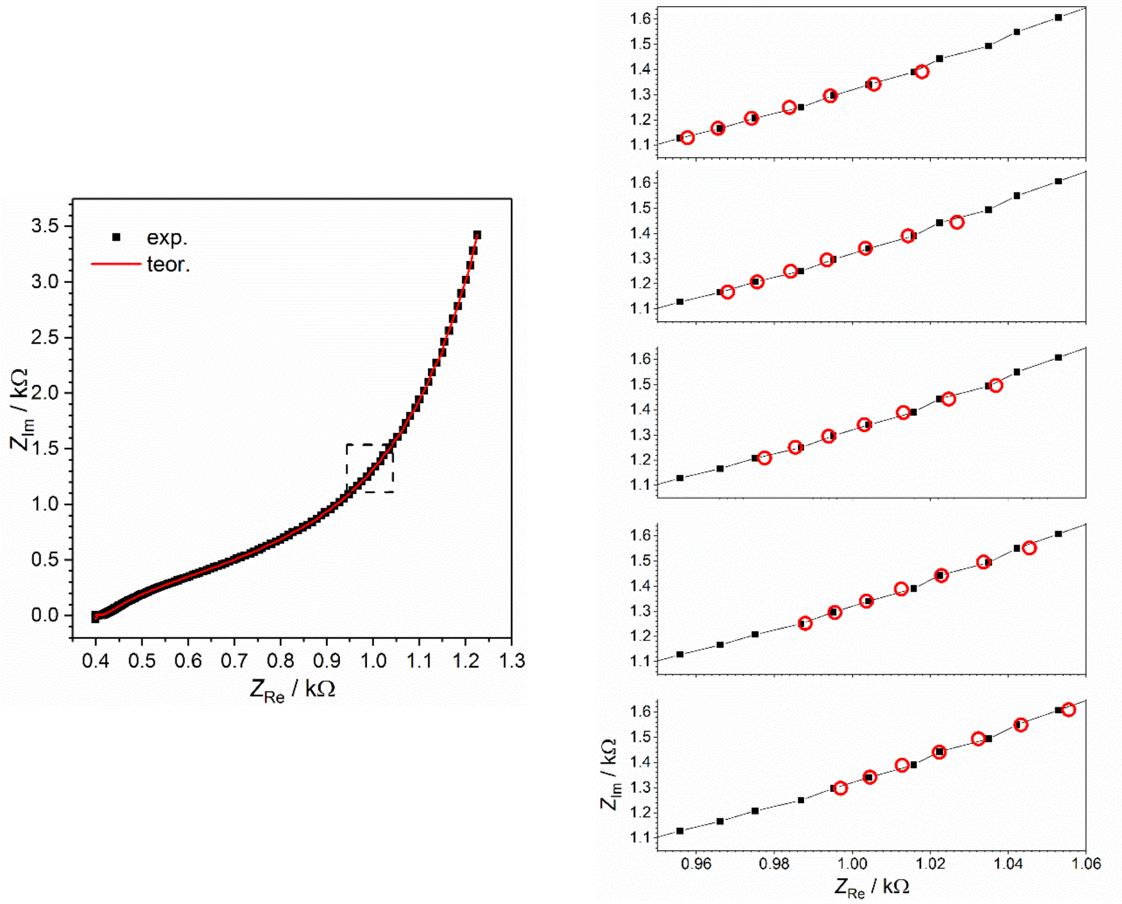

(a) (b)

Figure S7. Fragmentary analysis of the experimental data: fitting of the whole spectrum (**a**); fitting of the zoomed fragment (**b**).

A similar technique for fragmentary analysis has been already proposed by Stoynov et al. [3–5] in respect to the non-uniform research objects such as mesoporous oxide films. The general fit looks perfect (Figure S7a), because according to the current methodology each fragment is fitted individually. The selected fragments of the spectrum are zoomed to show the fitting quality. The calculated and experimental points fit satisfactorily, but not perfectly. The ideal fitting is not yet expected since these are experimental data. Otherwise, it would be suspicious and additional checks would be required. The selected number of points, seven, was found to be optimal. From the fitting quality point of view, it is about two times more than the number of varied parameters (equal 4). On the other hand, it covers a frequency range narrow enough to refer the data to one frequency.

In defence of our idea we remind that the conventional approach of fitting equivalent circuit in the whole range of frequencies could not be applied to the conductive polymer film, so was the reason to develop the advanced technique.

6. The Strategy for Extraction of Physical Parameter Values

According to our hypothesis, one branch of the EC (Figure S6b) is attributed to positively charged ions (cations) and the other to negatively charged ions (anions). Afterwards, the attributions will be confirmed by the careful analysis of the results. In the first surprise, for each spectrum one of the resistances appeared to be positive and the other to be negative, while both capacitances remained positive. At the early stages of the work, such results were regarded as erroneous, but their reproducibility in all the samples and under all conditions was convincing enough to accept them and think about explanations.

The answer on this paradox is hidden in Equation (S54). The only term that could be either positive or negative is *z* value in odd degree. The sign of *z* value defines the sign of *R*, whereas according to (S55), *C* is always positive, regardless of *z* due to the fact that $z^2 > 0$.

A careful look on the formulas would perceive that two equations contain four unknown variables (*c*, *D*, *E* and *d*), which makes the problem unsolvable. However, the experimental data for the research object contain manifold points.

The proposed strategy will be aimed at estimation of resistances and capacitances of each of the infinitesimal layers (Figure S3). The number of the layers to be estimated may be less than or equal to the number of frequency points, but not exceeding that number. Taking into account, that precision of the results requires minimisation of the thickness of the small layer, we will use the maximal possible number, i.e., the number that is equal to the number of frequency points. The procedure includes the following steps.

1. The initial approximate values of diffusion coefficients for cations (D_+) and anions (D_-) are proposed, e.g., $1\cdot10^{-10}$ $m^2\cdot s^{-1}$. The values will be further adjusted during the iteration steps.
2. The thickness of the polymer film is divided by *n* equal fragments ($\delta x = d/n$), *n* equals the number of the frequency.
3. The matrix $\Gamma_{nn} = [\gamma(\omega_i, x_j)]_{nn}$ is created. Each element is calculated by the formula (S56), which is formed from (S50) to estimate the average value of γ in the considered distance interval $[x_1; x_2]$.

$$\gamma(\omega,x)=\frac{1}{x_2-x_1}\int_{x_1}^{x_2} e^{-\sqrt{\frac{\omega}{2D}}x}\mathrm{d}x=\sqrt{\frac{2D}{\omega}}\frac{1}{x_2-x_1}\left(e^{-\sqrt{\frac{\omega}{2D}}x_1}-e^{-\sqrt{\frac{\omega}{2D}}x_2}\right) \quad \text{(s56)}$$

4. Two systems of equations have to be solved to estimate values of resistances and capacitances of each elementary layer ($R(x_i)$ and $C(x_i)$). The system of equation concerned with resistances is shown below in a matrix form: the full (S57a) and minor (S57b) version.

$$\begin{pmatrix} \gamma(\omega_1,x_1) & \gamma(\omega_1,x_2) & \dots & \gamma(\omega_1,x_n) \\ \gamma(\omega_2,x_1) & \gamma(\omega_2,x_2) & \dots & \gamma(\omega_2,x_n) \\ \dots & \dots & \dots & \dots \\ \gamma(\omega_n,x_1) & \gamma(\omega_n,x_2) & \dots & \gamma(\omega_n,x_n) \end{pmatrix} \times \begin{pmatrix} R(x_1) \\ R(x_2) \\ \dots \\ R(x_n) \end{pmatrix} = \begin{pmatrix} R(\omega_1) \\ R(\omega_2) \\ \dots \\ R(\omega_n) \end{pmatrix} \quad \text{(S57a)}$$

$$\Gamma_{nn}(\omega_i,x_j)\times \mathrm{R}_{n1}(x_j)=\mathrm{R}_{n1}(\omega_i) \quad \text{(S57b)}$$

The Γ-matrix has been calculated at the previous step, and the $R_{n1}(\omega_i)$-matrix represents the experimental data. The sought $R_{n1}(\omega_j)$-matrix is calculated by the formula (S58) using an inverse matrix approach.

$$\mathrm{R}_{n1}(x_j)=\Gamma_{nn}^{-1}(\omega_i,x_j)\times \mathrm{R}_{n1}(\omega_i) \quad \text{(S58)}$$

The same approach is applied to estimate values of capacitances.

5. Having the values of capacitances, resistances and diffusion coefficients at one's disposal, one can estimate the values of concentration in all the considered fragments.

$$c(x_i)=\frac{C(x_i)}{fF(\delta x)} \quad \text{(S59)}$$

$$E(x_i)=\frac{1}{zf^2FD\cdot c(x_i)\cdot R(x_i)} \quad \text{(S60)}$$

Although the proposed algorithm seems to be precise, its realisation required enormous accuracy and attention. The calculation of the inverse matrix in case of high-ranked matrices (more than 100) was a special problem, since a minor change in a single element caused recalculation of the whole mass of the data.

References

1 Láng, G.; Inzelt, G. An advanced model of the impedance of polymer film electrodes. *Electrochim. Acta* **1999**, *44*, 2037–2051, doi:10.1016/S0013-4686(98)00312-0.

2 Láng, G.; Ujvári, M.; Inzelt, G. Analysis of the impedance spectra of Pt|poly(o-phenylenediamine) electrodes - Hydrogen adsorption and the brush model of the polymer films. *J. Electroanal. Chem.* **2004**, *572*, 283–297, doi:10.1016/j.jelechem.2003.11.052.

3 Vladikova, D.; Stoynov, Z.; Viviani, M. Application of the differential impedance analysis for investigation of electroceramics. *J. Eur. Ceram. Soc.* **2004**, *24*, 1121–1127, doi:10.1016/S0955-2219(03)00585-5.

4 Vladikova, D.; Stoynov, Z. Secondary differential impedance analysis - A tool for recognition of CPE behavior. *J. Electroanal. Chem.* **2004**, *572*, 377–387, doi:10.1016/j.jelechem.2004.02.032.

5 Vladikova, D. Selectivity study of the differential impedance analysis—Comparison with the complex non-linear least-squares method. *Electrochim. Acta* **2002**, *47*, 2943–2951, doi:10.1016/S0013-4686(02)00187-1

© 2020 by the authors. Submitted for possible open access publication under the terms and conditions of the Creative Commons Attribution (CC BY) license (http://creativecommons.org/licenses/by/4.0/).
